# Surfactant Proteins A/D–CD14 on Alveolar Macrophages Is a Common Pathway Associated With Phagocytosis of Nanomaterials and Cytokine Production

**DOI:** 10.3389/fimmu.2021.758941

**Published:** 2021-10-27

**Authors:** Qiqi Wang, Qiong Wang, Ziyue Zhao, Jingbo Fan, Linghan Qin, David B. Alexander, Hiroyuki Tsuda, Dahai Zhao, Jiegou Xu

**Affiliations:** ^1^ Department of Immunology, School of Basic Medical Sciences, Anhui Medical University, Hefei, China; ^2^ Department of Clinical Laboratory, Anhui General Hospital of Chinese People’s Armed Police Forces, Hefei, China; ^3^ Department of Respiratory and Critical Care Medicine, The Second Affiliated Hospital, Anhui Medical University, Hefei, China; ^4^ Laboratory of Nanotoxicology Project, Nagoya City University, Nagoya, Japan

**Keywords:** engineered nanomaterials, alveolar macrophage, surfactant-associated protein A, surfactant-associated protein D, CD14, opsonization

## Abstract

Alveolar macrophages are responsible for clearance of airborne dust and pathogens. How they recognize and phagocytose a variety of engineered nanomaterials (ENMs) with different properties is an important issue for safety assessment of ENMs. Surfactant-associated proteins, specifically existing in the pulmonary surfactant, are important opsonins for phagocytosis of airborne microorganisms. The purposes of the current study are to understand whether opsonization of ENMs by surfactant-associated proteins promotes phagocytosis of ENMs and cytokine production, and to determine whether a common pathway for phagocytosis of ENMs with different properties exists. For these purposes, four ENMs, MWCNT-7, TiO_2_, SiO_2_, and fullerene C60, with different shapes, sizes, chemical compositions, and surface reactivities, were chosen for this study. Short-term pulmonary exposure to MWCNT-7, TiO_2_, SiO_2_, and C60 induced inflammation in the rat lung, and most of the administered ENMs were phagocytosed by alveolar macrophages. The ENMs were phagocytosed by isolated primary alveolar macrophages (PAMs) *in vitro*, and phagocytosis was enhanced by rat bronchioalveolar lavage fluid (BALF), suggesting that proteins in the BALF were associated with phagocytosis. Analysis of proteins bound to the 4 ENMs by LC/MS indicated that surfactant-associated proteins A and D (SP-A, SP-D) were common binding proteins for all the 4 ENMs. Both BALF and SP-A, but not SP-D, enhanced TNF-α production by MWCNT-7 treated PAMs; BALF, SP-A, and SP-D increased IL-1β production in TiO_2_ and SiO_2_ treated PAMs; and BALF, SP-A, and SP-D enhanced IL-6 production in C60 treated PAMs. Knockdown of CD14, a receptor for SP-A/D, significantly reduced phagocytosis of ENMs and SP-A-enhanced cytokine production by PAMs. These results indicate that SP-A/D can opsonize all the test ENMs and enhance phagocytosis of the ENMs by alveolar macrophages through CD14, suggesting that SP-A/D-CD14 is a common pathway mediating phagocytosis of ENMs. Cytokine production induced by ENMs, however, is dependent on the type of ENM that is phagocytosed. Our results demonstrate a dual role for surfactant proteins as opsonins for both microbes and for inhaled dusts and fibers, including ENMs, allowing macrophages to recognize and remove the vast majority of these particles, thereby, greatly lessening their toxicity in the lung.

## Introduction

The respiratory tract is the major exposure routine for airborne dusts and pathogens. Alveolar macrophages (AMs) reside in the airway and alveoli, and account for 95% of leukocytes in the lower respiratory tract (1). AMs function mainly in host defense and alveolar homeostasis. Phagocytosis by AMs is a major mechanism for clearance of dusts and microorganisms encountered in the lung (2). AMs have many types of pattern-recognition receptors (PRRs) on the plasma membrane, and recognize a variety of pathogen-associated molecular patterns (PAMPs) in microorganisms (3, 4). AMs also express receptors for the Fc portions of IgG antibodies (Fc receptors) and receptors for complement (complement receptors) that specifically bind IgG- or complement-coated pathogens (5, 6). The process of coating pathogens to promote phagocytosis is called opsonization. The direct binding of PRRs to their corresponding PAMPs and the binding of Fc receptors and complement receptors to opsonized pathogens induce a number of responses in AMs, including production of cytokines, inflammatory mediators and microbicidal enzymes, and mobilization of the cytoskeleton leading to phagocytosis, cell migration, and granule exocytosis. Secreted cytokines and inflammatory mediators then exert regulatory functions on inflammation and immune responses (7, 8). AMs are also involved in the clearance of apoptotic and necrotic cells and the subsequent resolution of pulmonary inflammation (9).

With advances in nanotechnology, engineered nanomaterials (ENMs) are increasingly being developed. The unique physicochemical features of ENMs including size effect, surface effect and quantum effect are very different from their bulk counterparts and make ENMs to be applied in almost every field such as material industry, semiconductor industry, daily life products and biomedical field. Increased production and application of ENMs may lead to increased respiratory exposure to ENMs during their production, consumption, and disposal. Understanding their pro-inflammatory and clearance mechanisms in the lung is critical for safety assessment of ENMs. Numerous studies in animals indicate that inhaled ENMs are taken up and cleared by AMs (10, 11). ENMs have little antigenicity and do not possess molecular structural regions like the PAMPs of microbes. How AMs recognize different ENMs with different sizes, chemical compositions, physical morphologies, and surface reactivity is currently an open question. It is widely accepted that ENMs are bound by proteins from various biological fluids, forming tiers of proteins coating the surface of the ENM. In turn, this protein corona may promote phagocytosis through interaction of bound complement proteins or IgG with complement receptors and Fc receptors (12, 13). The composition of the protein corona depends upon both the biological fluid in which the ENM is present and the physiochemical characteristics of the ENM (14). Most studies on the interaction between ENMs and proteins are based on protein coronas that form in serum/plasma. Pulmonary surfactant is a complex mixture composed of more than 90% lipids and 5-10% proteins that covers the inner face of the alveoli and reduces surface tension at the air-water interface in the alveoli to prevent alveolar collapse at the end of expiration (15). Surfactant-associated proteins (SP-A, SP-B, SP-C and SP-D) differ from one another in their synthesis, oligomerization, and function (16). SP-B and SP-C are hydrophobic and reduce the surface tension of the lung, while SP-A and SP-D are more hydrophilic and have important roles in the regulation of innate immune responses (17). SP-A and SP-D can bind to many different microbial pathogens and act as opsonins to enhance phagocytosis of microbial pathogens by AMs (18, 19). Coating of MWCNTs with pulmonary surfactant and bronchoalveolar lavage fluids (BALF)-derived protein corona on diesel exhaust nanoparticles enhance the nanoparticle uptake and pro-inflammatory responses in macrophages *in vitro* (20, 21), and SP-A and SP-D are found to bind the nanoparticles (21). Furthermore, SP-D can opsonize carbon nanotubes and augment their phagocytosis and pro-inflammatory responses *in vitro* (22). It is likely that surfactant proteins mediate phagocytosis of nanoparticles and subsequent cytokine production.

In the current study, we addressed two main questions: 1) whether a common spectrum of surfactant proteins was bound to different ENMs? 2) what proteins on AMs were responsible for recognizing the bound surfactant proteins and subsequent phagocytosis and cytokine secretion? For these purposes, 4 types of ENMs (fullerene C60, TiO_2_, SiO_2_, and MWCNT), differing in size, shape, surface reactivity, and chemical composition, were chosen for analysis of their binding proteins in rat bronchoalveolar lavage fluid (BALF) and cytokine productions. SP-A and SP-D were identified as common binding proteins for all 4 ENMs, and enhanced phagocytosis of the ENMs and cytokine production by AMs *in vitro*. Notably, the enhancing effects were dependent on CD14.

## Materials And Methods

### Preparation of Nanomaterial Suspensions

MWCNTs (MWCNT-7) were obtained from Mitsui Chemicals Co., Ltd. Tokyo, Japan; TiO_2_ (rutile type, with a mean primary size of 20 nm) was provided by Japan Cosmetic Association, Tokyo, Japan; SiO_2_ (with a primary size of 10-20 nm) was purchased from Sigma-Aldrich, USA; and Fullerene C60 (with a mean primary size of 1 nm) was provided by Frontier Carbon Corporation, Japan. 10 mg of the 4 types of ENMs was suspended in saline containing 0.5% (w/v) Pluronic^®^ F-68 (PF-68, a non-ionic detergent from Sigma-Aldrich, USA) to a final concentration of 500 μg/ml. The MWCNT-7 suspension was homogenized for 1 minute four times (total of 4 min) using a Polytron PT1600E bench-top homogenizer (Kinematica, Switzerland) at a speed of 3000 rpm. The prepared four nanomaterial suspensions were sonicated at 600W for 5 minutes 6 times with 2-minute rest intervals using a JY92-2 sonicator (Scientz Co., Ltd, Ningbo, China). To ensure the dispersion and suspension of the nanomaterials, the suspensions were further sonicated for 5 minutes 4 times before use. Characterization of the suspended 4 nanomaterials, including shape, element analysis, and size distribution are shown in **Supplementary Figures S1**–**S3**.

### Animals

Eight weeks-old female wild-type Sprague-Dawley (SD) rats were obtained from the Animal Center of Anhui Medical University. The rats were housed in the Animal Center and received Oriental MF basal diet and water *ad libitum*. The animal experiment protocols were approved by the Animal Ethics Committee of Anhui Medical University.

### Intratracheal Spraying of Nanomaterial Suspensions

Twenty-five female SD rats were divided in to 5 groups and about 0.5 ml of the vehicle or 500 μg/ml MWCNT-7, TiO_2_, SiO2, or C60 suspensions were administered by intratracheally spraying using an intratracheal aerosolizer (series IA-1B, Penn-century, Philadelphia, USA), as previously described (23), 2 times per week for 2 weeks. The total amount of the administered nanomaterials was 4 mg per kilogram of body weight for each rat. Three days after the last spraying, the animals were sacrificed under isoflurane anesthesia, and the lung was excised, fixed in 4% paraformaldehyde solution in phosphate-buffered saline (PBS) adjusted to pH 7.3, and processed for light microscopic examination and transmission electron microscopy (TEM) or scanning electron microscopy (SEM).

### Light Microscopy and Electron Microscopy

Hematoxylin-eosin (HE) stained sections of the lung tissues treated with the 4 ENM suspensions were used to observe lung inflammation and localization of the nanomaterials. For transmission electron microscopic (TEM) observation of TiO_2_, SiO_2_ and C60, paraffin blocks were deparaffinized and small pieces of the lung tissues were embedded in epon resin and processed for nanomaterial observation using a JEM-2100 transmission electron microscope (JEOL Co. Ltd, Tokyo, Japan). Since MWCNT-7 is difficult to be cut by an electronic microtome, scanning electron microscopy (SEM) was used to observe the MWCNT-7-treated lung tissues. Briefly, the HE-stained slides of the lung tissues were immersed in xylene for 3 days to remove the cover glass, immersed in 100% ethanol for 10 min to remove the xylene, and then air-dried for 2 hours at room temperature. The slides were then coated with platinum for observation using a Model S-4700 Field Emission SEM (Hitachi High Technologies Corporation, Tokyo, Japan) at 5–10 kV.

### Preparation of BALF and Isolation of Rat Alveolar Macrophages

Eight weeks-old female wild-type SD rats were placed under deep anesthesia with intraperitoneally injected sodium pentobarbital and sacrificed by exsanguination from the inferior vena cava. The lung was excised under aseptic conditions and injected with 5 ml of saline through the trachea. After gentle shaking, the fluid in the lung was collected. This was repeated 2 more times. The collected fluid was centrifuged at 1800g for 5min at 4°C, and the supernatant (BALF) was concentrated with a concentrator tube (Millipore) and stored at -80°C until use. The cell pellet was resuspended in RPMI 1640 medium containing 10% fetal bovine serum (Gibco, USA), seeded in a six-well plate and cultured at 37°C for 90 minutes. The cells were washed with PBS three times to remove red blood cells, other cells, and cell debris. The remaining adherent cells were stained with anti-CD68, a macrophage marker, to confirm their identity. Briefly, the adherent cells were fixed in 4% paraformaldehyde and treated with 0.2% Triton X-100 containing 10% fetal bovine serum (Gibco)/1% bovine serum albumin in PBS at room temperature for 15 minutes, and then incubated with rabbit anti-CD68 (1:50 dilution, Bioss, Beijing, China) overnight at 4°C, and visualized with Cy3 labelled anti-rabbit IgG (1:100 dilution, Proteintech, Wuhan, China). After washing, the cells were counter-stanning with DAPI (Sigma-Aldrich). Images were captured with a florescence microscope (ZEISS LSM880+Airyscan, Germany). As shown in **Supplementary Figure S4**, more than 95% of the adherent cells were positive for CD68. About 5×10^5^-1x10^6^ alveolar macrophages per rat were isolated.

### Binding of Nanomaterials to BALF Proteins, SDS-PAGE, and LC-MS

1.6 ml of the four ENM suspensions (equal to 800 μg of each ENM) were incubated with 1 ml of the 20-fold concentrated rat BALF at 37°C in a shaker at 200 rpm for 4-6 hours, and then centrifuged at 20000×g for 30 minutes to separate the nanomaterials from the supernatants. The precipitates were re-suspended in PBS, and the suspensions were centrifuged at 20000×g for 10 minutes and the supernatants were discarded. The washing steps were repeated 2 more times. The final ENM precipitates with their bound proteins were resuspended in 50 μl of sodium dodecyl sulfate polyacrylamide gel electrophoresis (SDS-PAGE) buffer (10% glycerol, 62.5 mM Tris-HCl [pH 6.8], 2% 2-mercaptoethanol, 2% SDS), heated for 5 minutes at 95°C, and then centrifuged at 20000×g for 30 minutes to dissociate the bound proteins from the nanomaterial precipitates. After protein quantification with BCA (BestBio, Shanghai, China), 10 μl of the final suspensions was subjected for SDS-PAGE and the gels was silver-stained with a silver staining kit (BestBio). DTT was added to 20 μl of the remaining SDS-PAGE buffer-dissociated protein solution to a final concentration of 100 mM DTT and boiled 5 minutes. The samples were analyzed by Liquid Chromatography with Tandem Mass Spectrometry (LC-MS) (Shanghai Applied Protein Technology Company, Ltd, Shanghai, China).

### Exposure of ENMs to Primary Alveolar Macrophages

*In vitro* 1×10^6^ rat primary alveolar macrophages (PAMs) were cultured at 37°C overnight in a 6-well plate in RPMI 1640 culture medium containing 10% fetal bovine serum (Gibco). The PAMs were then washed 3 times with PBS and cultured in X-VIVO™ serum-free medium (Lonza, Belgium) The cells were then treated with saline containing 0.5% (w/v) Pluronic^®^ F-68, 1 μg/ml MWCNT-7, TiO_2_, SiO2, or C60 suspended in saline containing 0.5% (w/v) Pluronic^®^ F-68, or 1 μg/ml of MWCNT-7, TiO_2_, SiO2, or C60, pre-incubated for 4 hours with 1 μg/ml of recombinant human SP-A, SP-D or 10% concentrated BALF, and continued culture for 12 hours. The cells were harvested for RNA isolation and quantitative polymerase chain reaction (qPCR) analysis of cytokine expression, and the culture supernatants were collected for ELISA detection of cytokines.

### qPCR and ELISA Analyses of IL-1β, IL-6, and TNF-α Production

Total RNA from rat PAMs was extracted with Trizol (Magen, Guangzhou, China) according to the manufacturer’s instructions. 500 ng of the RNA samples was reverse transcribed using a HiScript® II RT SuperMix kit (Vazyme Biotech, Nanjing, China), and qPCR analyses of IL-1β, IL-6, and TNF-α were then performed using the AceQqPCR SYBR Green Master Mix (Vazyme). GAPDH was used as an internal reference, and the relative expression of each gene was analyzed by the 2 ^-△△CT^ method. The primer pairs (forward/backward) used were the following: CAGCAGCATCTCGACAAGAG/CATCATCCCACGAGTCACAG for IL-1β; AGTT- GCCTTCTTGGGACTGA/TCCAAGATCTCCCTGAGAACA for IL-6; ACTCCCAGA- AAAGCAAGCAA/CGAGCAGGAATGAGAAGAGG for TNF-α; and GACATGCCG- CCTGGAGAAAC/AGCCCAGGATGCCCTTTAGT for GAPDH. Detection of IL-1β, IL-6, and TNF-α in the supernatants of rat primary alveolar macrophage cultures was performed with rat ELISA kits (MLBio, Shanghai, China) according to the manufacturer’s instructions.

### Analysis of ENM Phagocytosis by Polarized Light Microscopy

1×10^6^ rat PAMs were seeded in 6-cm culture dishes with pre-placed microscope cover glass (NEST, China) and cultured in X-VIVO™ serum-free medium (Lonza) and cultured overnight. The cells were treated with saline containing 0.5% (w/v) Pluronic^®^ F-68; 1 μg/ml MWCNT-7, TiO_2_, SiO2, or C60 suspended in saline containing 0.5% (w/v) Pluronic^®^ F-68; or 1 μg/ml of MWCNT-7, TiO_2_, SiO2, or C60, pre-incubated for 4 hours with 1 μg/ml of recombinant human SP-A, SP-D or 10% concentrated BALF, and continued culture for 12 hours. The cells were then fixed with 4% paraformaldehyde and stained with HE. The Cells with phagocytosed ENMs were identified using an ECLIPSE polarizing microscope (LV100NPOL, Nikon, Japan). Total cell number and the number of the cells with phagocytosed ENMs in 10 fields (40x magnification) were counted, and the percent of the cells with phagocytosed ENMs (number of cells with phagocytosed ENMs/total number of cells) was determined.

### Knockdown of LRP1, CD14, and SIRPα and Its Effect on Cytokine Production and Phagocytosis

Small interfering RNAs (siRNA) were used to knock-down the expression of LDL receptor related protein 1 (LRP1), CD14, and signal regulatory protein alpha (SIRPα). 3 pairs of siRNAs for each of the genes were provided by GenePharma Co. Ltd., Shanghai, China. The pair of siRNAs with the best silencing efficacy was determined by preliminary experiments. The siRNA sequences used for further experiments were as follows: GCUAAACUCGCUCAAUCUATT/UAGAUUGAGCGAGUUUAGCTT for CD14; CCAUCAAACGGGCAUUCAUTT/AUGAAUGCCCGUUUGAUGGTT for LRP1; and GCUCUAUGUACUCGCCAAATT/UUUGGCGAGUACAUAGAGCTT for SIRPα. 1×10^6^ rat PAMs were seeded in each well of a 6-well plate and cultured at 37°C overnight. Negative control RNA or siRNAs for LRP1, CD14, and SIRPα were transfected into the cells using Lipofectamine 2000 (Thermo Fisher, USA). 6 hours later, the culture media was changed to X-VIVO™ serum-free medium (Lonza), and then treated with 1 μg/ml MWCNT-7, TiO_2_, SiO2, or C60 suspended in saline containing 0.5% (w/v) PF-68; or 1 μg/ml of MWCNT-7, TiO_2_, SiO2, or C60, pre-incubated for 4 hours with 1 μg/ml of recombinant human SP-A, SP-D or 10% concentrated BALF, and continued culture for 12 hours. The cells were harvested for RNA isolation, qPCR analysis of silencing efficacy and cytokine expression, and western blotting; the culture supernatants were collected for ELISA. The silencing efficacy of LRP1, CD14, and SIRPα was analyzed by qPCR, as described above, and confirmed by western blotting. The specific primers used were CCAGGAACTTTGGCTTTGCTC/ACCGATGGACAACTTTCAGG for CD14; CCAATTGTGCATTTTTGCAG/GAATCAGGGGCATAGGTGAA for LRP1; and GTGTCTGTTGCTGCTGGAGA/GCATCTTCTGGGGTGACATT for SIRPα. The expression of LRP1, CD14, and SIRPα proteins was detected by western blotting. The cells were lysed in RIPA buffer (150mM NaCl, 50mM Tris pH 7.4, 1% sodium deoxycholate, 0.1% SDS, 1% Triton X-100, and 1mM PMSF) for 5 minutes and centrifuged at 4°C 12000 rpm for 20min. After protein quantification with a BCA kit (Bestbio), aliquots of the supernatants (20 μg protein) were separated by 10% sodium dodecyl sulfate polyacrylamide gel electrophoresis (SDS-PAGE) and then transferred to a PVDF membrane (Millipore, Boston, USA). After blocking in 5% nonfat milk, the PVDF membranes were incubated with primary antibodies (anti-GAPDH from Peprotech, USA, 1:500 dilution, as an internal control; anti-CD14 from Bioss, Beijing, China, 1:500 dilution; anti-SIRPα from Cell Signaling Technology, Danvers, USA, 1:1000 dilution; anti-LRP1 from Abcam, USA, 1:10000 dilution) at 4°C overnight. After three washings, the PVDF membrane was incubated with peroxidase conjugated anti-mouse or anti-rabbit secondary antibodies (1:10,000) for 60 minutes. The protein was visualized with ECL (Thermo Fisher) detection solution in a GEL Imaging System (Tanon, Shanghai, China). The effect of LRP1, CD1, and SIRPα knockdown on IL-1β, IL-6, and TNF-α production was analyzed by qPCR and ELISA, and its effect on ENM phagocytosis was assessed by polarized light microscopy, as described above.

### Statistical Analysis

Statistical analysis was performed using SSPS17 software. The statistical significance was analyzed using two tailed Student’s t test. A p value of <0.05 was considered to be significant.

## Results

### Most Intratracheally Administered Nanomaterials Were Taken up By Alveolar Macrophages

Four types of ENMs with different shapes, chemical compositions, sizes, and surface properties were chosen for this study. MWCNT-7 is a carbon-based nanotube with a hydrophobic surface; TiO_2_ is a rod-like particle with a hydrophilic surface; SiO_2_ and C60 are round nanoparticles with a hydrophilic and hydrophobic surface, respectively. They form aggregates with different sizes in suspensions. Characterization of their shape and chemical composition is shown in **Supplementary Figures S1, S2**. Their size distribution of the suspensions in 0.5% PF-68 saline was comparable to that of the suspensions in 0.5% PF-68 saline supplemented with the concentrated BALF (**Supplementary Figure S3**).

**Figure 1:**
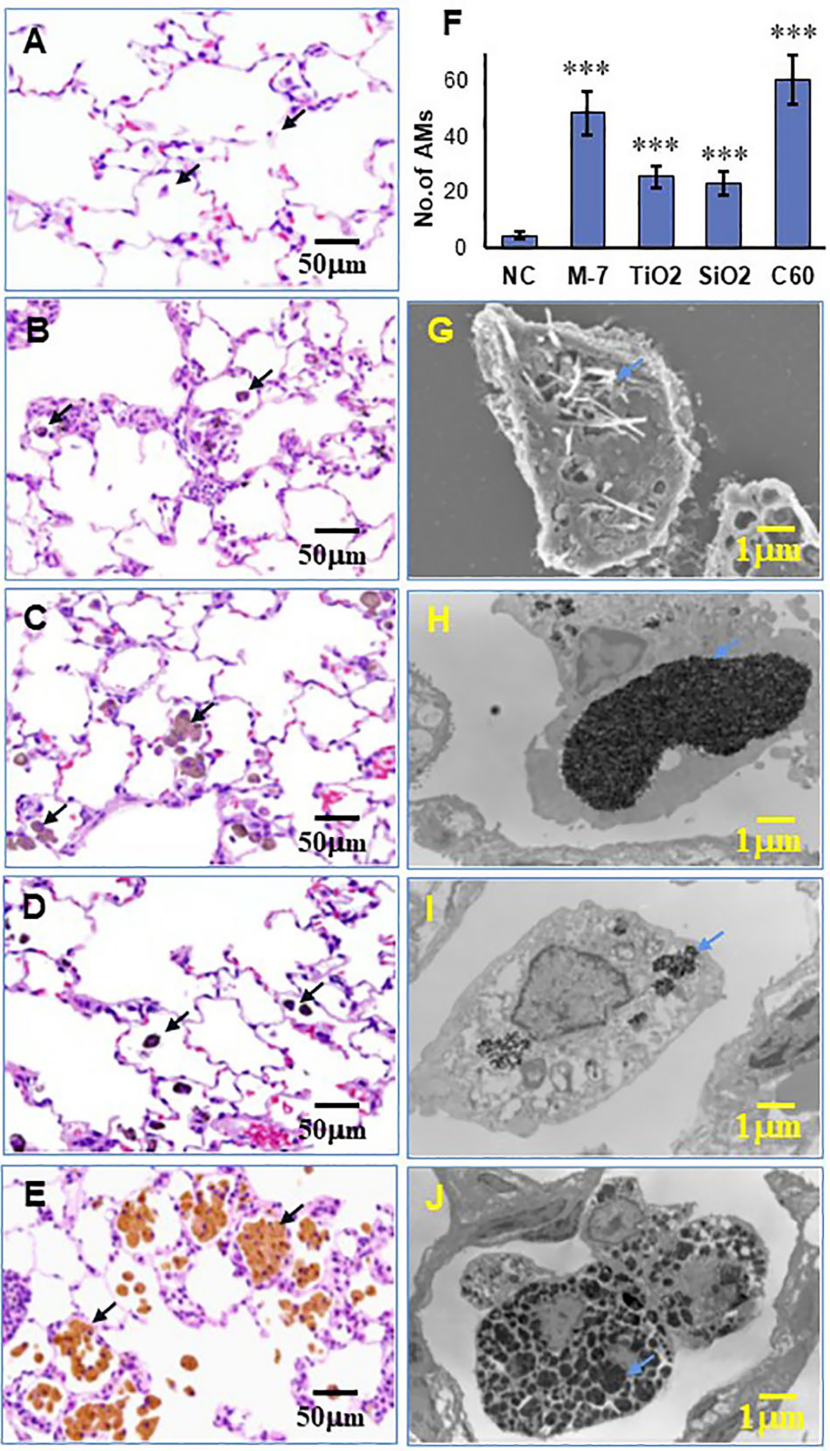
Lung inflammation and phagocytosis of ENMs by alveolar macrophages. Female SD rats were intratracheally sprayed with 0.5 ml of 500 μg/ml MWCNT-7, TiO_2_, SiO_2_, or C60 suspensions. Lung inflammation in HE-stained slides was examined under light microscopy, and alveolar macrophages with phagocytosed ENMs were indicated with black arrows in the left panel. Representative HE images treated with the control, MWCNT-7, TiO2, SiO2, or C60 were shown in **(A–E)**, respectively. The number of alveolar macrophages per 200x sight field in the treated lung tissues was presented in **(F)**. Phagocytosis of MWCNT-7 in **(G)** was observed under scanning electron microscopy, while the phagocytosed TiO2, SiO2, or C60 in **(H–J)**, respectively, were observed under transmission electron microscopy. The blue arrows in the right panel indicated the phagocytosed ENMs, and *** represents p values less than 0.001.

Intratracheal spraying is a simple method for delivery of agents into the lung. In our preliminary experiment, black ink administered into the lung by intratracheal spraying was delivered evenly to each lobe and found to be taken up by AMs (**Supplementary Figure S4**). Intratracheal administration of MWCNT-7, TiO_2_, SiO_2_, and C60 suspensions to the rat lung induced pulmonary inflammation to different extents. Compared with 0.5% PF-68 saline (NC, **Figure 1A**), MWCNT-7 elicited a strong inflammatory response, with increased number of AMs and thickening of the alveolar epithelium (**Figure 1B**); while TiO_2_ and SiO_2_ (**Figures 1C, D**) had little effect on the alveolar epithelium, although particle-burdened alveolar macrophages were often observed. Similar to MWCNT-7, C60 caused a strong inflammatory response (**Figure 1E**). The number of AMs per 200◊sight field in the MWCNT-7, TiO_2_, SiO_2_, or C60 treated lung tissues was significantly increased compared with that of the NC (**Figure 1F**). Most of the induced AMs were found with burden ENM aggregates (**Figures 1B**–**E**). Further observation with SEM for MWCNT-7 (since MWCNT-7 is difficult to be cut by an electronic microtome) and TEM for TiO_2_, SiO_2_, and C60 revealed that the ENM aggregates were in the cytoplasm of the AMs and likely to exist in the phagosomes (**Figures 1G**–**J** correspondingly for MWCNT-7, TiO_2_, SiO_2_, and C60). These results indicate that uptake by AMs is a main mechanism for clearance of the deposited particles.

### BALF Enhanced the Uptake of Nanomaterials by Alveolar Macrophages

**Figure 2:**
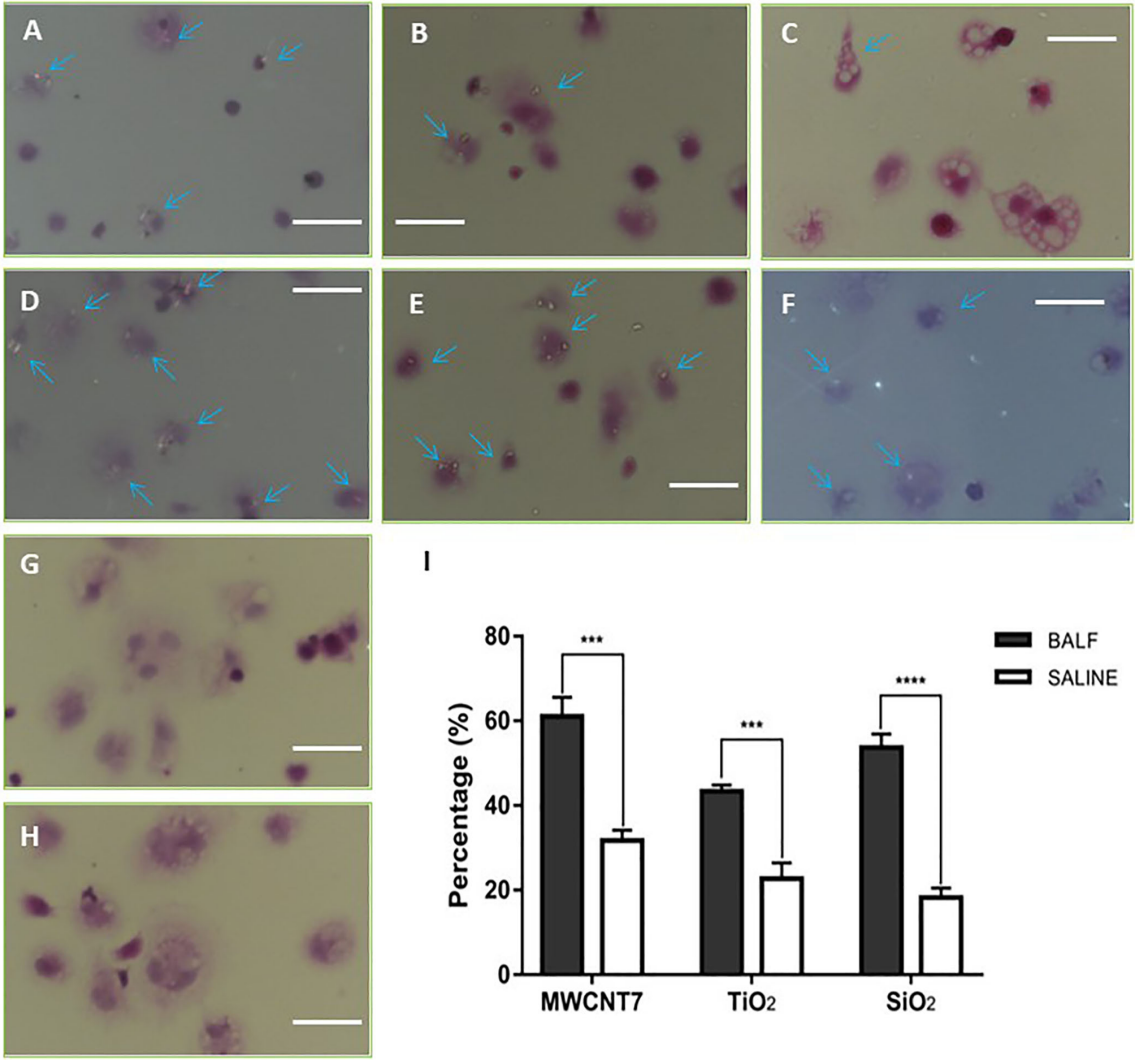
Influence of BALF on phagocytosis of ENMs by primary alveolar macrophages. Primary alveolar macrophages were exposed *in vitro* to 4 types of ENMs (MWCNT-7, TiO_2_, SiO_2_, and C60) in the absence **(A–C, G)** or presence **(D–F, H)** of BALF. Phagocytosis of ENMs was examined by polarized light microscopy under 40×manification. **(A, D)**, specimens from an MWCNT-7 treated rat, **(B, E)**, specimens from a TiO_2_ treated rat. **(C, F)**, specimens from an SiO_2_ treated rat. **(G, H)** specimens from a C60 treated rat. Arrows indicate alveolar macrophages with phagocytosed ENMs; bars = 50 μm. C60 is isotropic, and therefore, C60 cannot be specifically identified by polarized microscopy. Consequently, only percentages of alveolar macrophages with phagocytosed MWCNT-7, TiO_2_, and SiO_2_ were calculated **(I)**. *** and **** represent p values less than 0.001 and 0.0001, respectively.

Previous studies have demonstrated that ENMs are bound by proteins biological fluids, forming tiers of proteins surrounding the nanomaterials (14). This protein corona affects the biological behavior of the nanomaterial, including phagocytosis by macrophages (24). To determine whether secretory fluid in the respiratory tract and alveoli influences phagocytosis by alveolar macrophages of the 4 ENMs being studied, we prepared BALF and primary alveolar macrophages (PAMs). Immunofluorescence staining indicated that more than 95% of isolated cells were positive for CD68, a macrophage marker (**Supplementary Figure S5**). The 4 ENMs, with or without pre-incubation of BALF, were then exposed *in vitro* to the isolated PAMs. The number of PAMs with internalized ENMs with or without pre-incubation of BALF were compared under polarized light microscopy. The results indicated that pre-incubation with BALF increased the uptake of MWCNT-7, TiO_2_, and SiO_2_ by PAMs (**Figure 2**). The percent of PAMs with particles without pre-incubation of BALF (31.7%, 22.7%, and 18.2% for MWCNT-7, TiO_2_, and SiO_2_, respectively) was significantly lower than with pre-incubation of BALF (61.0%, 43.3%, and 53.7% for MWCNT-7, TiO_2_, and SiO_2_, respectively) (**Figure 2I**). Because C60 is isotropic and cannot be specifically visualized using polarized light microscopy, therefore, phagocytosis of C60 was not quantified (**Figures 2G, H**). The results obtained from MWCNT-7, TiO_2_, and SiO_2_ suggest that proteins in the BALF are likely to adsorb to ENMs and promote their uptake by PAMs.

Usually, there are two ways for internalization of ENMs into a cell: clathrin-dependent endocytosis and actin-dependent phagocytosis. For determining whether the 4 ENMs were taken up *via* phagocytosis, PAMs were treated with cytochalasin B (an inhibitor of actin polymerization, thus inhibiting phagocytosis) and exposed with MWCNT-7, TiO_2_, and SiO_2_ pre-incubated with BALF. Pre-treatment of cytochalasin B significantly reduced the percentage of the PAMs with burden-particles (**Supplementary Figure S6**), indicating that uptake of the ENMs by AMs is mainly *via* phagocytosis.

### SP-A, SP-D, and SP-B Are Common Proteins Associated With ENMs

**Figure 3:**
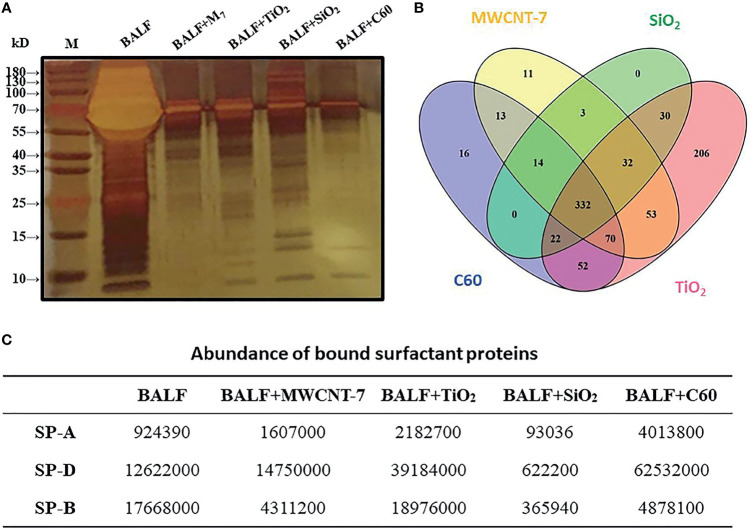
*Analysis of ENM-bound proteins.* 800 μg of MWCNT-7, TiO_2_, SiO_2_, or C60 was incubated with 1 ml of 20-fold concentrated rat BALF at 37°C for 4-6 hr. The bound proteins were then disassociated from the ENMs and analyzed by SDS-PAGE and LC/MS. **(A)** imagine of SDS-PAGE; **(B)** Venn diagram showing the distribution of the proteins bound to the 4 ENMs; and **(C)** abundance of SP-A, SP-D, and SP-B bound to each type of ENM.

For understanding possible interactions of the proteins present in the BALF with the 4 ENMs, the ENMs were incubated in 20-fold concentrated BALF, precipitated by centrifugation, washed with PBS, and resuspended in SDS-PAGE buffer. The ENM associated proteins were then subjected to SDS-PAGE. SDS-PAGE and silver staining revealed that proteins with different molecular weights and abundance absorbed to the surfaces of the 4 ENMs (**Figure 3A**). Further analysis with LC-MS identified a total of 854 proteins associated with the 4 ENMs. 332 of these proteins were found to be associated with each of the 4 ENMs (**Figure 3B**). The 120 most abundant proteins associated with each of the 4 ENMs are shown in **Supplementary Table S1**. Most of these proteins were serum-derived proteins commonly found in BALF, including albumin, complement proteins, immunoglobulins, and apolipoproteins. Lung/respiratory tract derived proteins were also common.

Pulmonary surfactant is a complex mixture containing 4 surfactant-associated proteins (SP-A, SP-B, SP-C and SP-D). Notably, SP-A, SP-B, and SP-D were found to be associated with all 4 ENMs (**Figure 3C**). SP-D was among the most abundant proteins associated with MWCNT-7, TiO_2_, SiO_2_, and C60, while SP-B was among the most abundant proteins associated with MWCNT-7, SiO_2_, and C60 (**Supplementary Table S1**).

For further confirming that SP-A and SP-D were bound proteins, the 4 ENMs were incubated with 10-fold concentrated BALF and then separated by centrifugation. Immunofluorescence showed that the large aggregates in the precipitates were stained with anti-SP-A and anti-SP-D (**Supplementary Figure S7A**) and ELISA detection revealed that concentrations of SP-A and SP-D in the supernatants were decreased by the incubation with the 4 ENMs (**Supplementary Figure S7B**), demonstrating that SP-A and SP-D were bound to the 4 ENMs.

### SP-A and SP-D Enhanced Nanomaterial-Induced Macrophage Activation and Cytokine Production

**Figure 4:**
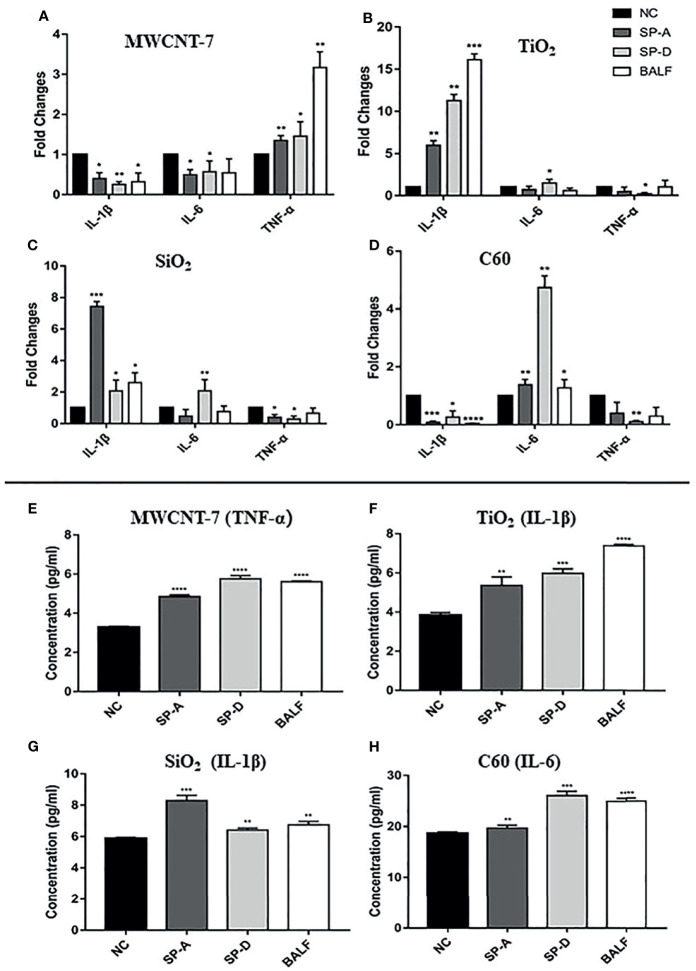
Effects of SP-A and SP-D on cytokine production. Rat primary alveolar macrophages in X-VIVO™ serum-free medium were treated with the vehicle; or 1 μg/ml MWCNT-7, TiO_2_, SiO_2_, or C60; or 1 μg/ml MWCNT-7, TiO_2_, SiO_2_, or C60 pre-incubated with 1 μg/ml of recombinant human SP-A, SP-D, or 10% concentrated BALF, and cultured for 12 hours. The RNAs isolated from the treated cells were analyzed by qPCR for expression of TNF-α, IL-1β and IL-6 **(A–D)**. The culture media was collected and used for ELISA detection of TNF-α, IL-1β, and IL-6 **(E–H)**. **(A, E)** treatment with MWCNT-7. **(B, F)**, treatment with TiO_2_. **(C, G)**, treatment with SiO_2_.D and H, treatment with C60. *, **, *** and **** p values less than 0.05, 0.01, 0.001, and 0.0001, respectively, compared with the negative control (NC).

We initially investigated the effect of SP-A, SP-B, and SP-D on macrophage activation, using TNF-α, IL-1β and IL-6, the main pro-inflammatory cytokines produced upon macrophage activation, as markers of macrophage activation. Preliminary experiments showed that SP-B-coated ENMs had little effect on the production of these cytokines (data not shown). Similarly, SP-A, SP-D, or BALF alone in the absence of the ENMs (**Supplementary Figure S8A**), or each of the 4 ENMs alone without SP-A or SP-D coating (**Supplementary Figure S8B**) did not significantly affect cytokine production as detected by qPCR. In contrast, MWCNT-7, pre-incubated with 1μg/ml human recombinant SP-A or SP-D, stimulated a significant increase in TNF-α mRNA expression of PAMs, but not IL-1β or IL-6 mRNA expression (**Figure 4A**). Similar results were also observed when MWCNT-7 was pre-incubated with BALF (**Figure 4A**). Examination of the culture media by ELISA indicated that pre-incubation with SP-A, SP-D, and BALF enhanced MWCNT-7-induced TNF-α secretion (**Figure 4E**).

In TiO_2_ treated PAMS, pre-incubation with SP-A or BALF enhanced IL-1β expression, while pre-incubation with SP-D enhanced both IL-1β and IL-6 expression (**Figure 4B**). Similarly, ELISA showed increased IL-1β secretion in the PAMs treated with TiO_2_ that was pre-incubated with SP-A, SP-D, or BALF (**Figure 4F**). The response of SiO_2_ treated PAMS was similar to that of TiO_2_ treated PAMS: SP-A and BALF enhanced IL-1β expression and SP-D enhanced both IL-1β and IL-6 expression (**Figure 4C**). ELISA showed increased IL-1β secretion in the presence of SP-A, SP-D, and BALF (**Figure 4G**). Treatment withC60 pre-incubated with SP-A, SP-D, or BALF upregulated IL-6 expression at both the mRNA and protein secretion, as detected by qPCR and ELISA (**Figures 4H, D**).

In brief, the effect of SP-A and SP-D on macrophage activation as assessed by TNF-α, IL-1β, and IL-6 expression was similar to that of BALF. However, the specific effect on cytokine expression depended on the ENM to which the PAMs were exposed.

### Knockdown of CD14 Expression in Alveolar Macrophages Reduced Cytokine Production

**Figure 5:**
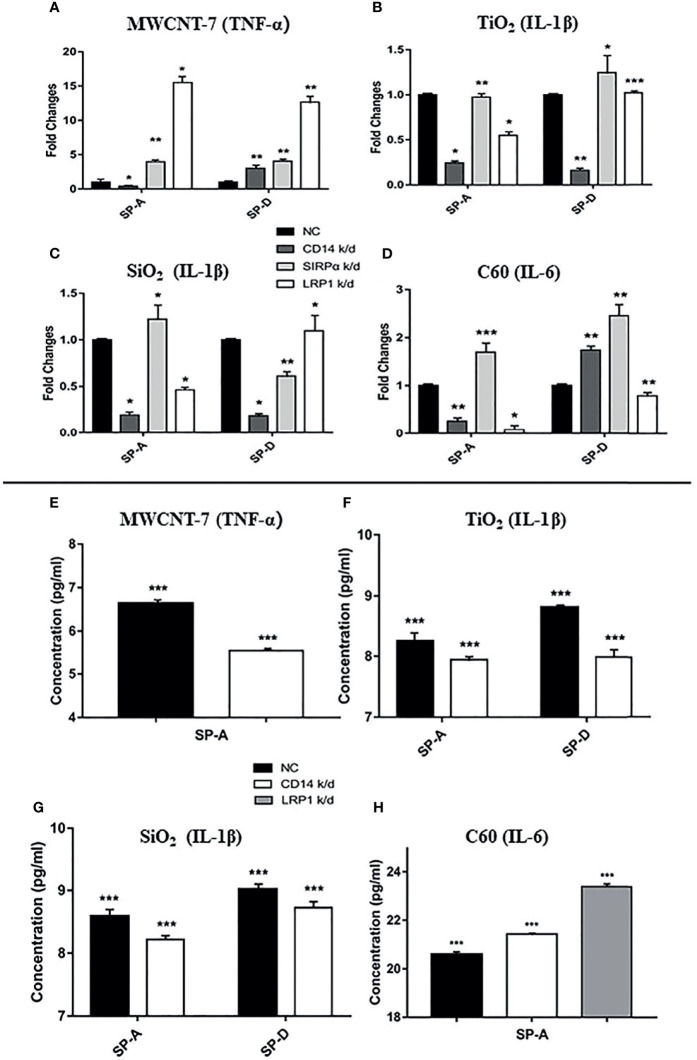
Effect of LRP1, CD14, and SIRPα knockdown on cytokine production. Expression of LRP1, CD14, and SIRPα in rat primary alveolar macrophages was knocked down (k/d) by gene-specific siRNAs. Macrophages were then treated with the vehicle; or 1 μg/ml MWCNT-7, TiO_2_, SiO_2_, or C60; or 1 μg/ml MWCNT-7, TiO_2_, SiO_2_, or C60 pre-incubated with 1 μg/ml of recombinant human SP-A, SP-D, or 10% concentrated BALF, and cultured for 12 hours. The cells were harvested for RNA isolation and qPCR analysis **(A–D)** and the culture supernatants were collected for ELISA **(E–H)**. **(A, E)** detection of TNF-α in MWCNT treated cells; **(B, F)** detection of IL-1β in TiO_2_ treated cells; **(C, G)** detection of IL-1β in SiO_2_ treated cells; and **(D, H)** detection of IL-6 in C60-treated cells. *, ** and *** p values less than 0.05, 0.01, and 0.001, respectively, compared with the negative control (NC).

It has been reported that LRP1, CD14, and SIRPα are potential receptors for SP-A and SP-D (25–27). Thus, we used siRNA to knock-down the expression of these receptors (**Supplementary Figures S9A, B**). Knockdown of LRP1, CD14, or SIRPα did not significantly change mRNA expression of the cytokines in PAMs (**Figure S10A**). Similarly, neither addition of SP-A or SP-D into the culture medium, nor stimulation with each of the ENMs, affected the cytokine expression at the mRNA level in the receptors-knocked down PAMs (**Supplementary Figures S10B, C**). However, Knockdown of CD14 significantly reduced SP-A-enhanced TNF-α expression in MWCNT-7 stimulated PAMs (**Figures 5A, E**), while SP-D-enhanced TNF-α expression was increased by CD14 silencing (**Figure 5A**). TNF-α expression was also elevated by knockdown of LRP1 and SIRPα (**Figure 5A**). The marked increase in SP-A and SP-D enhanced TNF-α expression by knockdown of LRP1 suggests that LRP1 may be inhibitory to TNF-α production by PAMs.

Knockdown of CD14 decreased SP-A- or SP-D-enhanced IL-1β expression in TiO_2_- and SiO_2_-stimulated PAMs (**Figures 5B, C** **, F, G**). Silencing of CD14 expression also decreased SP-A-enhanced IL-6 expression by C60 stimulated PAMs (**Figures 5D, H**). Knockdown of LRP1 also markedly decreased IL-6 mRNA expression in C60 stimulated PAMs (**Figure 5D**). However, in contrast to mRNA expression, CD14 and LRP1 knockdown increased IL-6 protein secretion by C60 stimulated PAMS. Overall, knockdown of CD14 consistently downregulated SP-A enhanced cytokine mRNA expression, suggesting a common SP-A-CD14 axis for ENM-induced cytokine production.

### Knockdown of CD14 Expression in Alveolar Macrophages Reduced ENM Uptake

**Figure 6:**
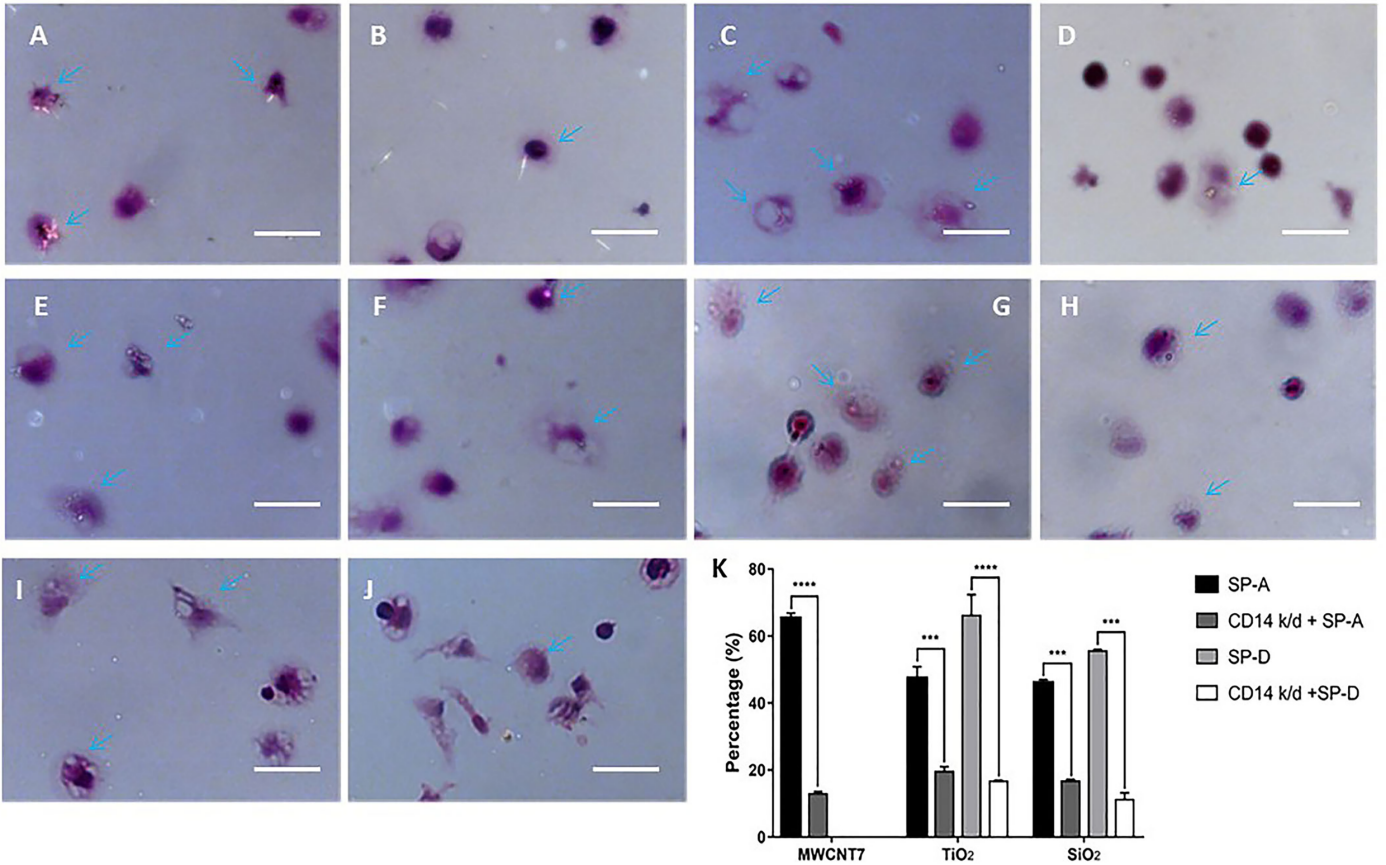
Effect of CD14 knockdown on phagocytosis of MWCNT-7, TiO_2_, and SiO_2_. Polarized light microscope images of primary alveolar macrophages treated with MWCNT-7 pre-incubated with SP-A **(A)**; CD14-knockdown primary alveolar macrophages treated MWCNT-7 pre-incubated with SP-A **(B)**; primary alveolar macrophages treated with TiO_2_ pre-incubated with SP-A **(C)**; CD14-knockdown primary alveolar macrophages treated with TiO_2_ pre-incubated with SP-A **(D)**; primary alveolar macrophages treated with TiO_2_ pre-incubated with SP-D **(E)**; CD14-knockdown primary alveolar macrophages treated with TiO_2_ pre-incubated with SP-A **(F)**; primary alveolar macrophages treated with SiO_2_ pre-incubated with SP-A **(G)**; CD14-knockdown primary alveolar macrophages treated with SiO_2_ pre-incubated with SP-A **(H)**; primary alveolar macrophages treated with SiO_2_ pre-incubated with SP-D **(I)**; and CD14-knockdown primary alveolar macrophages treated with SiO_2_ pre-incubated with SP-D **(J)**. Percentages of alveolar macrophages with phagocytosed MWCNT-7, TiO_2_, and SiO_2_ were shown **(K)**. Arrows indicate alveolar macrophages with phagocytosed ENMs; bars = 50 μm. *** and **** represent p values less than 0.001 and 0.0001, respectively.

Finally, we determined the effect of CD14 knockdown on the phagocytosis of ENMs by PAMs. CD14 knockdown reduced phagocytosis of MWCNT-7 pre-incubated with SP-A by PAMs: With pre-incubation of SP-A, 65.3% of the observed PAMS contained MWCNT-7 fibers while only 12.9% of CD14 knockdown PAMs contained MWCNT-7 fibers (**Figures 6A, B, K**). CD14 knockdown also reduced phagocytosis of TiO_2_ and SiO_2_: In the presence of SP-A, 47.3% of the PAMs contained TiO_2_ particles while only 19.5% of CD14 knockdown PAMs contained TiO_2_ particles (**Figures 6C, D, K**); in the presence of SP-D, 66.1% of PAMs contained TiO_2_ particles while only 16.6% of CD14 knockdown PAMs contained TiO_2_ particles (**Figures 6E, F, K**); in the presence of SP-A, 46.0% of the PAMs contained SiO_2_ particles while only 16.7% of CD14 knockdown PAMs contained TiO_2_ particles (**Figures 6G, H, K**); in the presence of SP-D, 55.5% of PAMs contained SiO_2_ particles while only 11.2% of CD14 knockdown PAMs contained TiO_2_ particles (**Figures 6I, J, K**). As noted above, because C60 is isotropic, phagocytosis of C60 could not be evaluated using polarized light microscopy.

## Discussions

Pulmonary inflammation is a common lesion by inhaled ENMs and other airborne dusts. The extent and type of pulmonary inflammation depend on the amount and type of inhaled ENMs deposited in the lung particular in the alveolar region, since mucociliary movement in the upper respiratory tract rapidly and effectively clears large particles deposited in the upper airways. Because of their tiny size, ENMs tend to deposit in the alveolar region. ENMs deposited in the alveolar region are gradually eliminated over time by various clearance mechanisms. If ENMs cannot be effectively cleared, persistent chronic inflammation may lead to chronic obstructive pulmonary disease, emphysema and lung fibrosis. In this study, we chose 4 types of ENMs (MWCNT, TiO_2_, SiO_2_ and C60), each with different shapes, chemical compositions, sizes, and surface properties, and tried to find out a common clearance mechanism and different cytokine production by alveolar macrophages (AMs). The results may contribute to their toxicological safety assessment and provide instruction for production of safe ENMs.

AMs phagocytose and clear airborne dusts and microbial pathogens encountered in the lung. Inhaled ENMs are also cleared from the lung by macrophages. ENMs have relatively simple chemical compositions. Thus, unlike microorganisms composed of biological macromolecules, most types of ENMs do not have structures that are recognized by receptors, such as PRRs expressed on macrophages. Opsonization of ENMs by proteins that are recognized by macrophages is a likely mechanism by which macrophages recognize and phagocytose ENMs. However, while it is known that SP-A and SP-D are found in the corona of PEG-, PLGA-, or Lipid-modified nanoparticles (28) and that SP-A increases cellular binding and uptake of nanoparticles coated with different polymers by AMs (29), the process by which alveolar macrophages recognize and phagocytose uncoated ENMs has not yet been characterized. The present study was undertaken to investigate this question.

We initially examined the effect of bronchioalveolar lavage fluid (BALF) on the phagocytosis of 3 different types of ENMs (MWCNT-7, TiO_2_, and SiO_2_) and found that BALF enhanced phagocytosis of these ENMs by primary rat AMs: C60, the fourth type of ENM used in our study, is isotropic and therefore specific phagocytosis of C60 could not be observed by polarized light microscopy. Next, we examined the proteins associated with the ENMs that could be involved in phagocytosis of these particles. We identified 854 proteins that co-precipitated with the 4 different types of ENMs. Of the 854 proteins associated with these ENMs, 332 proteins were associated with all 4 ENMs. Notably, the lung surfactant proteins SP-A, SP-B, and SP-D were associated with all 4 ENMs, and SP-B and SP-D were among the most abundant proteins associated with MWCNT-7, SiO2, and C60. We therefore tested the effect of SP-B, SP-D, and SP-A on macrophage activation by MWCNT-7, TiO_2_, SiO_2_, and C60. SP-B did not affect macrophage activation by any of the 4 test ENMs. In contrast, SP-A and SP-D enhanced macrophage activation by all 4 test ENMs. These results are in agreement with the known functions of these proteins: SP-B and SP-C are hydrophobic and reduce the surface tension of the lung, while SP-A and SP-D are more hydrophilic and act as opsonins to enhance phagocytosis of microbial pathogens by AMs (18, 19).

Next, we determined the effect of LRP1, CD14, and SIRPα knockdown on the production of cytokines by AMs exposed to the test ENMs in the presence of SP-A or SP-D. While our experiments were performed using primary rat AMs rather than cell lines, allowing variation in CD14 expression levels to exist between cells, downregulation of CD14 in populations of AMs markedly reduced SP-A/ENM-induction of TNF-α mRNA expression by MWCNT-7 treated AMs, IL-1β mRNA expression by TiO_2_ and SiO_2_ treated AMs, and IL-6 expression by C60 treated AMs, and to a lesser extent, CD14 knockdown decreased protein expression of these cytokines in MWCNT-7, TiO_2_, and SiO_2_ treated cells. Importantly, downregulation of CD14 also markedly reduced phagocytosis. These results indicate the involvement of the SP-A-CD14 pathway in the phagocytosis of ENMs and subsequent activation of AMs. In the presence of SP-D, downregulation of CD14 also reduced L-1β mRNA expression by TiO_2_ and SiO_2_ treated AMs, but did not reduce TNF-α mRNA expression by MWCNT-7 treated AMs or IL-6 expression by C60 treated AMs. This result suggests that the SP-D-CD14 pathway differs from the SP-A-CD14 pathway. Since both SP-A and SP-D were associated with all 4 ENMs and CD14 is a receptor for both SP-A and SP-D, the result with SP-D appears to conflict with the result with SP-A. However, the difference is likely related to the fact that CD14 binds to SP-A and SP-D *via* different mechanisms [22], and that the protein corona surrounding the different ENMs is highly complex [14], making it is likely that the interaction and orientation of SP-A and SP-D with ENMs are dependent on the type of ENM to which they attach (summarized in **Supplementary Figure S11**).

SP-A and SP-D are lung-specific proteins existing in the pulmonary surfactant. In addition to their functions in the homeostasis of the pulmonary surfactant (30, 31), they are also involved in the host defense against various pulmonary pathogens, such as respiratory syncytial virus, mycobacterium tuberculosis, bacteria, viruses and fungi (32, 33). Structurally, SP-A and SP-D belong to the collectin family with a C-terminal carbohydrate recognition domain (CRD) and an N-terminal collagen like domain (34) and act as opsonins by interaction *via* the CRD with various microorganisms and their derived components to enhance phagocytic function of AMs through CD14, Toll-like receptors and other receptors expressing on the surface of AMs (22, 35). Our results, indicate that opsonization by SP-A and/or SP-D is an important defense mechanism not only in the elimination of invading microbes, but also in the clearance of inhaled ENMs and is likely to be important in the clearance of other dusts as well. Our results also suggest that other proteins found in pulmonary surfactant, such as IgG and complement proteins, may also be involved in the clearance of ENMs from the lung, since they were also found to bind to MWCNT-7, TiO_2_, SiO_2_ and C60 (**Supplementary Table S1**).

Another finding of the current study is that different ENMs induced the expression of different cytokines: increased expression of TNF-α by MWCNT-7; increased expression of IL-1β by TiO_2_ and SiO_2_; and increased expression of IL-6 by C60. The difference in the induction of these cytokines, and other cytokines, likely contributed to the difference and extent of the pulmonary inflammation in ENM treated rats (see **Figure 1**). For example, MWCNT-7 and C60, which enhance expression of TNF-α and IL-6, respectively, by activated macrophages, are much more inflammatory than TiO_2_ and SiO_2_, which enhance expression of IL-1β: while IL-1β is a well know inflammatory cytokine, it can also contribute to the resolution of inflammation (36). However, in chronic inflammatory conditions, IL-1β can promote carcinogenesis (37). Thus, while the primary factor in the lung toxicity of an ENM is bio-persistence in the lung, the type of cytokines expressed by activated macrophages also affects the lung toxicity of inhaled ENMs.

In conclusion, SP-A and SP-D opsonized all four of the studied ENMs. SP-A enhanced phagocytosis of the 3 ENMs (MWCNT-7, TiO_2_, SiO_2_) that could be observed by polarized light microscopy, and SP-A enhanced macrophage activation by all 4 ENMs, indicating that SP-A also enhanced phagocytosis of C60. SP-D also enhanced phagocytosis and macrophage activation by TiO_2_ and SiO_2_. Interaction of SP-A and SP-D opsonized ENMs with macrophages was *via* interaction with CD14. Our results demonstrate a role for SP-A and SP-D as opsonins for all the test ENMs, allowing macrophages to recognize and remove the vast majority of these particles, thereby, greatly lessening their toxicity in the lung. Our results also show that expression of cytokines by ENM-activated macrophages can differ depending on the type of ENM that comes into contact with the macrophage. However, detailed molecular mechanisms for ENM-specific cytokine induction need further investigation. Importantly, such studies need to include ENMs treated with the biological fluids, such as BALF, present in the tissue in which interactions with the test ENMs occur.

## Data Availability Statement

The original contributions presented in the study are included in the article/**Supplementary Material**. Further inquiries can be directed to the corresponding authors.

## Ethics Statement 

The animal study was reviewed and approved by Animal Ethics Committee of Anhui Medical University.

## Author Contributions

QQW, QW, ZZ, JF, and LQ were responsible for the experiments. QQW and QW analyzed the data. QW wrote the initial manuscript, and DA and JX revised the manuscript. HT, DZ, and JX designed the study and provided the funding. All authors contributed to the article and approved the submitted version.

## Funding

This study was supported by the Key Research and Development Project of Anhui Province (201904b11020024 and 201904a07020064), Health and Labor Sciences Research Grants of Japan (Research on Risk of Chemical Substance 21340601, H25-kagaku-ippan-004 and H24-kagaku-sitei-009), Foundation of Education Bureau of Anhui Province China (KJ2016SD29), and Hefei Municipal Natural Science Foundation (2021037).

## Conflict of Interest

The authors declare that the research was conducted in the absence of any commercial or financial relationships that could be construed as a potential conflict of interest.

## Publisher’s Note

All claims expressed in this article are solely those of the authors and do not necessarily represent those of their affiliated organizations, or those of the publisher, the editors and the reviewers. Any product that may be evaluated in this article, or claim that may be made by its manufacturer, is not guaranteed or endorsed by the publisher.
