# Supplementary material for: Surfactant Proteins A/D–CD14 on Alveolar Macrophages Is a Common Pathway Associated With Phagocytosis of Nanomaterials and Cytokine Production
Source: Front Immunol. 2021 Oct 27;12:758941. doi: 10.3389/fimmu.2021.758941 (PMC8578846; doi:10.3389/fimmu.2021.758941)
Supplement: Supplementary file 1 [file DataSheet_1.pdf]

Supplementary Material

This supplementary material contains 11 figures and 1 table:

Figure S1 Shapes of MWCNT-7, TiO_2, SiO_2 and C60 in the suspension;

Figure S2 Chemical composition of MWCNT-7, TiO_2, SiO_2 and C60;

Figure S3 Size distribution of MWCNT-7, TiO_2, SiO_2 and C60 in the suspension;

Figure S4 Intratracheal spraying;

Figure S5 Immunostaining of primary alveolar macrophages;

Figure S6 Uptake of ENMs by PAMs via phagocytosis;

Figure S7 Binding of SP-A and SP-D to the ENMs;

Figure S8 Effects of SP-A, SD-D, BALF and ENMs on expression of cytokines;

Figure S9 Knockdown of CD14, LRP1, and SIRPα expression in PAMs;

Figure S10 Effects of SP-A, SD-D, BALF and ENMs on expression of cytokines in the receptors-knocked down PAMs;

Figure S11 Summary of the results;

Table S1 LC-MS analysis of the nanomaterial-bound proteins.

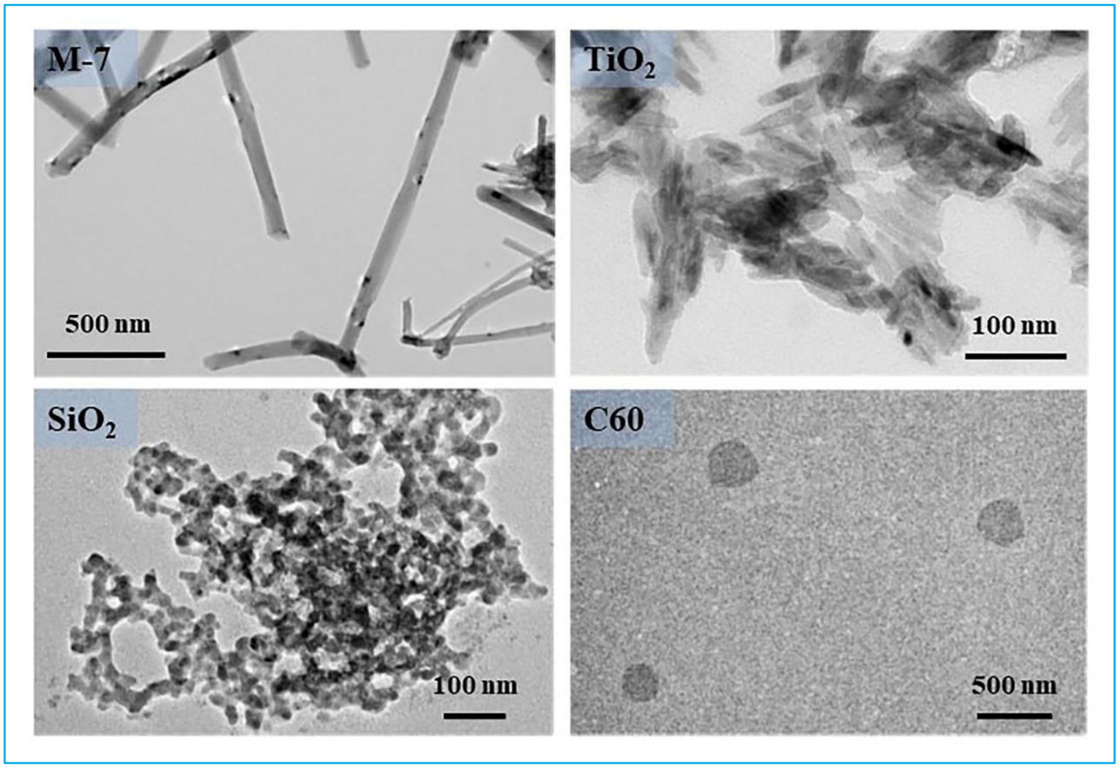

Figure S1 Shapes of MWCNT-7, TiO_2, SIO_2, and C60 in the suspension

MWCNT-7, TiO_2, SiO_2, and C60 were suspended in saline containing 0.5% (w/v) Pluronic® F-68 to a final concentration of 500 μg/ml. Suspensions were diluted with ddH_2O 1:200 and placed on a carbon sheet. After air-drying, the samples were observed under the JEM-2100 transmission electron microscope (JEOL Co. Ltd, Tokyo, Japan).

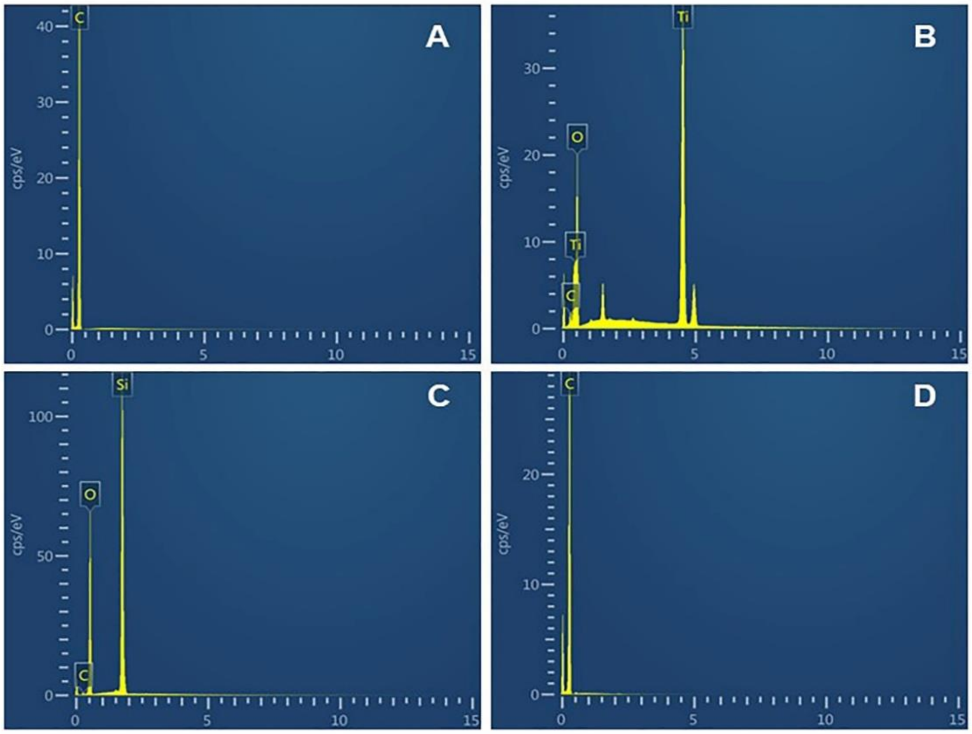

Element analysis (weight %)

Element	M7	TiO_2	SiO_2	C60
C	100.00	0.74	1.96	100.00
O		37.36	49.71	
Ti		61.90		
Si			48.33	

Figure S2 Chemical composition of MWCNT-7, TIO_2, SIO_2, and C60s

Suspensions of MWCNT-7, TiO_2, SiO_2, and C60 (500 μg/ml) were diluted with ddH_2O 1:200 and placed on an aluminum plate. After air-drying, the samples were observed and analyzed by FIB-SEM (Helios G4 UC, Aztec) connected with an EBSD analysis system (Thermo Scientific). A, B, C, and D represent the spectra of MWCNT-7, TiO_2, SiO_2, and C60, respectively. The table under the spectra is the element composition and ratio of each of the ENMs.

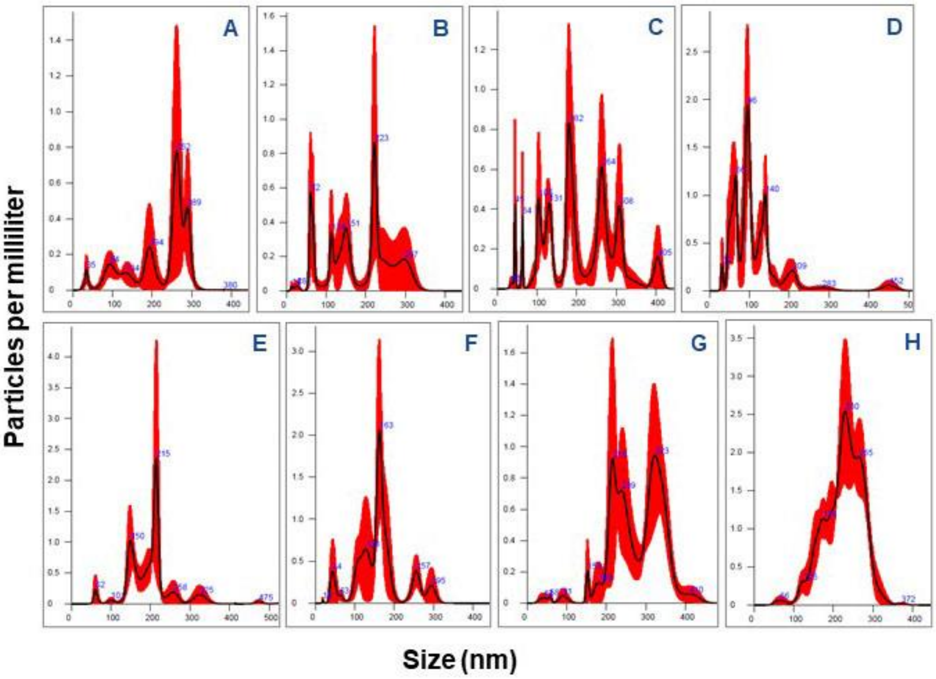

E Analysis of size in the suspension (mean ± s.d. nm)

ENM	W/O BALF	With BALF
M7	187.4±78.2	220.4±73.1
TiO_2	181.3±88.9	115.6±71.4
SiO_2	206.2±76.0	162.1±57.9
C60	225.7±48.0	277.6±65.9

Figure S3 Size distribution of MWCNT-7, TIO_2, SIO_2, and C60 in the suspension

1.6 ml of the four ENM suspensions (equal to 800 µg of each ENM) were added with 1 ml of saline or the 20-fold concentrated rat BALF, and incubated at 37℃ in a shaker at 200 rpm for 4-6 hours. Size distribution in 500 µg/ml suspensions of MWCNT-7, TiO_2, SiO_2 and C60 in the absence (A, C, E and G) or presence (B, D, F and H) of BALF were analyzed with a NanoSight 300 analyzer (Malvern). The representative size distribution curves are shown in figures A and B for MWCNT-7, C and D for TiO_2, E and F for SiO_2, and G and H for C60, respectively. The mean size and standard deviation (s.d.) for each kind of the ENMs are displayed in the table (E)

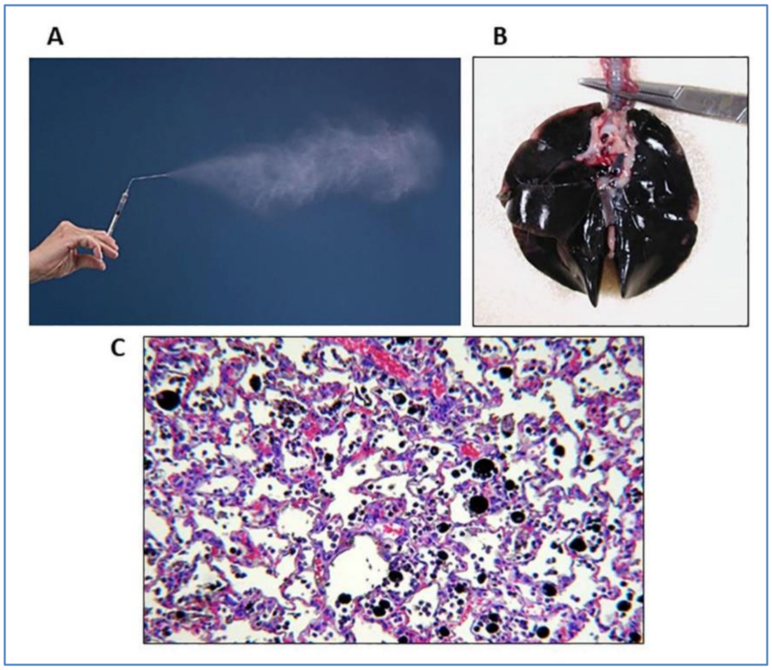

Figure S4 Intratracheal spraying

1.0 ml of black ink was sprayed into the lung of a 6-week-old SD rat using an intratracheal aerosolizer (series IA-1B, Penn-century, Philadelphia, USA). A. the intratracheal aerosolizer; B. the sprayed ink was evenly distributed in each lobe of the lung; and C. light microscopy showed the sprayed ink in the lung tissue.

Table S1 LC-MS analysis of the nanomaterial-bound proteins

The bound proteins in each group were analyzed by LC/MS. The top 120 proteins in the table are listed by values of IBAQ (intensity-based absolute-protein-quantification) indicating abundance of each bound protein.

No.	MWCNT-7	TiO2	SiO2	C60
1	Hemoglobin subunit beta-2	Hemoglobin subunit alpha-1/2	Glyceraldehyde-3-phosphate [illegible]	Hemoglobin subunit beta-2
2	Hemoglobin subunit alpha-1/2	Hemoglobin subunit beta-2	Hemoglobin subunit beta-1	Beta-actin
3	Beta-actin	Uncharacterized protein	Elongation factor 1-alpha	Hemoglobin subunit alpha-1/2
4	Glyceraldehyde-3-phosphate [illegible]	Hemoglobin subunit beta-1	Beta-actin	Fibrinogen beta chain
5	Apolipoprotein A-II	Beta-actin	Hemoglobin subunit alpha-1/2	Anionic trypsin-1
6	Serum albumin	Elongation factor 1-alpha	Protein S100-A8	Fibrinogen gamma chain
7	Uncharacterized protein	Glyceraldehyde-3-phosphate dehydrogenase	Apolipoprotein A-I	Glyceraldehyde-3-phosphate dehydrogenase
8	Apolipoprotein C-II	Uncharacterized protein	Eukaryotic translation initiation factor [illegible]	Fibrinogen alpha chain
9	Uteroglobin	Protein S100-A6	Uncharacterized protein	Uncharacterized protein
10	Elongation factor 1-alpha	LOC367586 protein	T-complex protein 1 subunit beta	Beta-defensin
11	Apolipoprotein C-I	Histidine-rich glycoprotein	Apolipoprotein C-III	Elongation factor 1-alpha
12	Hemoglobin subunit beta-1	Protein S100-A9	Hemoglobin subunit beta-2	Apolipoprotein A-II
13	Heat shock 27kDa protein 1	Lysozyme	Transketolase	Myosin light polypeptide 6
14	LOC367586 protein	Apolipoprotein H	Apolipoprotein E	Hemoglobin subunit beta-1
15	Beta-defensin 4	Apolipoprotein A-II	Histidine-rich glycoprotein	Lysozyme
16	Protein S100	Protein S100-A8	Tubulin beta chain	Heat shock protein beta-1

17	Lysozyme	Apolipoprotein E	Apolipoprotein A-IV	**Pulmonary surfactant-associated protein D**
18	BPI fold-containing family A member 1	Tubulin polymerization-promoting protein family member 3	Apolipoprotein C-I	Myosin regulatory light chain 12B
19	Apolipoprotein A-I	Ig gamma-2B chain C region	Lysozyme	Apolipoprotein C-II
20	Apolipoprotein C-III	Zero beta-globin	LOC367586 protein	Myosin, heavy polypeptide 9
21	Glutathione peroxidase 3	Four and a half LIM domains 1	Serum albumin	Tubulin beta-4B chain
22	Fibrinogen alpha chain	Ig kappa chain C region	Lipoprotein lipase	Apolipoprotein A-I
23	Tubulin beta chain	Annexin A1	Heat shock 27kDa protein 1	Apolipoprotein C-I
24	Fibrinogen gamma chain	Heat shock 27kDa protein 1	Adenylyl cyclase-associated protein 1	Apolipoprotein E
25	Uncharacterized protein	Beta-defensin 4	Beta-defensin 4	Uteroglobin
26	ADP-ribosylation factor 5	Igh-6 protein	Apolipoprotein A-II	Protein S100-A6
27	Fibrinogen beta chain	Transketolase	Complement C4	Globin c2
28	Ig gamma-2B chain C region	GTP-binding nuclear protein Ran	Uteroglobin	LOC367586 protein
29	Zero beta-globin	Fibrinogen gamma chain	Transketolase	Fibronectin
30	Pneumo secretory protein 1	Complement component C9	Transthyretin	Apolipoprotein A-IV
31	Clusterin	Tubulin beta chain	BPI fold-containing family A member 1	Serum albumin
32	ADP-ribosylation factor 3	Globin a4	Fibrinogen beta chain	BPI fold-containing family A member 1
33	Ig lambda-2 chain C region	Uncharacterized protein	ADP-ribosylation factor 5	Ribosomal protein S19-like2
34	Uncharacterized protein	Fibrinogen beta chain	Apolipoprotein C-II	Dermcidin
35	Alpha-2-HS-glycoprotein	Apolipoprotein C-I	Heat shock cognate 71 kDa protein	Zero beta-globin
36	Keratin, type I cytoskeletal	Fibrinogen alpha chain	Ig gamma-2B chain C region	Kng2 protein
37	Myosin light polypeptide 6	Eukaryotic translation initiation factor 5A-1	Apolipoprotein H	ADP-ribosylation factor 5
38	Inter alpha-trypsin inhibitor, heavy chain 4	Globin c2	Fibrinogen alpha chain	GTP-binding nuclear protein

39	Apolipoprotein A-IV	Apolipoprotein A-IV	Fibrinogen gamma chain	Ig kappa chain C region
40	Serotransferrin	Coagulation factor XII	Four and a half LIM domains 1	Barrier-to-autointegration factor
41	Apolipoprotein E	Cofilin-1	Beta-2 globin	Lymphocyte antigen 6B
42	Kng2 protein	Actin, alpha cardiac muscle 1	Serine protease inhibitor	Histone cluster 1 H1 family member
43	Ribosomal protein S19-like2	Apolipoprotein A-I	Inter alpha-trypsin inhibitor, heavy chain 4	Apolipoprotein C-III
44	Ras-related C3 botulinum toxin substrate [illegible]	Peroxiredoxin-2	Adiponectin c	Uncharacterized protein
45	Cell division control protein 42 homolog	Vitronectin	ADP-ribosylation factor 3	Clusterin
46	Tubulin beta-5 chain	Myosin light polypeptide 6	Myosin light polypeptide 6	Uncharacterized protein
47	BWK3	Peroxiredoxin-4	Actin, alpha cardiac muscle 1	40S ribosomal protein S3
48	**Pulmonary surfactant-associated protein D**	Apolipoprotein C-II	40S ribosomal protein S12	Uncharacterized protein
49	40S ribosomal protein S3	Complement C4	Uncharacterized protein	RAT Igh-6 protein
50	Transforming protein RhoA	Complement inhibitory factor H	Tubulin polymerization-promoting protein family member 3	Glutathione peroxidase 3
51	40S ribosomal protein S12	Ig lambda-2 chain C region	Dermcidin	Transforming protein RhoA
52	Uncharacterized protein	40S ribosomal protein S12	Complement inhibitory factor H	Tissue-type transglutaminase
53	Ig kappa chain C region	Protein disulfide-isomerase	Elongation factor 2	RAT Ig gamma-2B chain C region
54	Serine/cysteine peptidase inhibitor, clade C, member 1	40S ribosomal protein S3	Peroxiredoxin-2	BWK3
55	Histone cluster 1 H1 family member d	Serine protease inhibitor	Translationally-controlled tumor protein	High mobility group box 1
56	40S ribosomal protein S4	Cysteine and glycine-rich protein 1	Carbamoyl-phosphate synthetase 2, aspartate transcarbamylase, and	Annexin A2
57	Platelet factor 4	Ezrin	Protein S100-A6	Ras-related protein Rap-1b
58	GTP-binding nuclear protein Ran	C-type lectin domain family 3, member B	Complement component C9	Septin-9
59	40S ribosomal protein S14	Kng2 protein	Ribosomal protein S19-like2	Protein S100-A11
60	Ras-related protein Rap-1b	Elongation factor 2	GTP-binding nuclear protein Ran	Ig lambda-2 chain C region

61	Myosin, heavy polypeptide 9	Ig gamma-2C chain C region	Histone H4	Pneumo secretory protein 1
62	Actin, alpha cardiac muscle 1	Heat shock protein HSP 90-alpha	Tubulin beta-5 chain	Similar to 60S acidic ribosomal protein P2
63	SAR1 gene homolog A	BPI fold-containing family A member 1	C-type lectin domain family 3, member B	Tubulin beta-5 chain
64	BPI fold-containing family B member 1	Ras-related C3 botulinum toxin substrate 1	Myosin regulatory light chain 12B	40S ribosomal protein S14
65	Hemopexin	Ribosomal protein S19-like2	**Pulmonary surfactant-associated protein D**	60S ribosomal protein L22
66	Transthyretin	Transforming protein RhoA	Ras-related C3 botulinum toxin substrate 1	ATP-binding cassette sub-family B member 9
67	Igh-6 protein	Translationally-controlled tumor protein	Pneumo secretory protein 1	Platelet factor 4
68	Poly(rC)-binding protein 2	Heat shock cognate 71 kDa protein	40S ribosomal protein S3	Protein S100-A8
69	60S ribosomal protein L11	Inter alpha-trypsin inhibitor, heavy chain [illegible]	Ig kappa chain C region	Plasminogen
70	Destrin	Heat shock protein HSP 90-beta	Clusterin	T-complex protein 1 subunit beta
71	RAB1A, member RAS oncogene family	Myosin regulatory light chain 12B	Moesin	Alpha-2-HS-glycoprotein
72	Uncharacterized protein	Protein S100-A11	Kng2 protein	40S ribosomal protein S6
73	Cofilin-1	Adenylyl cyclase-associated protein 1	Igh-6 protein	Secretoglobin, family 3A, member 1
74	Uncharacterized protein	Glutathione peroxidase 3	Ras-related protein Rap-1b	RAB1A, member RAS oncogene family
75	RAB5C, member RAS oncogene family	Serum albumin	Uncharacterized protein	Annexin A1
76	Glutathione peroxidase 1	Uncharacterized protein	Transforming protein RhoA	Calcium-binding protein 4
77	40S ribosomal protein S5	40S ribosomal protein S18	Desmoplakin	Peroxiredoxin-1
78	Dermcidin	Apolipoprotein C-III	Uncharacterized protein	Cell division control protein 42 homolog
79	Heat shock cognate 71 kDa protein	Cysteine-rich protein 1	Glutathione peroxidase 3	40S ribosomal protein S3a
80	40S ribosomal protein S7	Complement factor B	Anti-F4/80 kappa light chain variable [illegible]	Myosin light chain 1/3
81	40S ribosomal protein S3a	Fibronectin	Globin c2	40S ribosomal protein S12
82	Uncharacterized protein	Eukaryotic translation initiation factor 1	Uncharacterized protein	Histidine-rich glycoprotein

83	Annexin A1	Annexin A2	Proliferation-associated protein 2G4	SAR1 gene homolog A
84	Galectin-1	Uncharacterized protein	Uncharacterized protein	Actin, alpha cardiac muscle 1
85	60S ribosomal protein L22	Uncharacterized protein	Fibronectin	Uncharacterized protein
86	Heat shock protein HSP 90-alpha	Moesin	Cysteine and glycine-rich protein 1	Cytochrome b5
87	Heat shock protein HSP 90-beta	BWK3	Annexin A1	Ras-related C3 botulinum toxin substrate 1
88	Protein S100-A11	Uncharacterized protein	Alpha-2-HS-glycoprotein	40S ribosomal protein S17-like
89	Prothrombin	ADP-ribosylation factor 5	Histone cluster 1 H1 family member d	Myosin heavy chain 14
90	Heterogeneous nuclear ribonucleoproteins A2/B1	Uncharacterized protein	Heat shock protein HSP 90-alpha	40S ribosomal protein S18
91	Myosin regulatory light chain 12B	Dynein light chain 1	GTP-binding protein SAR1b	ADP-ribosylation factor 3
92	Kininogen-1	Inter-alpha-trypsin inhibitor heavy chain 2	Uncharacterized protein	Ras-related protein Rab-6A
93	Uncharacterized protein	Uteroglobin	Heat shock protein HSP 90-beta	ADP-ribosylation factor 4
94	Secretoglobin, family 3A, member 1	Adiponectin	**Pulmonary surfactant-associated protein B**	Globin a4
95	Uncharacterized protein	Uncharacterized protein	TNF alpha-induced protein 8	40S ribosomal protein S5
96	Immunoglobulin joining chain	Complement C3	Myosin, heavy polypeptide 9	60S ribosomal protein L11
97	ADP-ribosylation factor 4	Myristoylated alanine-rich C-kinase substrate	40S ribosomal protein S4	40S ribosomal protein S4
98	40S ribosomal protein S6	EH domain-containing protein 1	Cofilin-1	Glutathione peroxidase 1
99	Uncharacterized protein	40S ribosomal protein S14	Uncharacterized protein	**Pulmonary surfactant-associated protein B**
100	40S ribosomal protein S25	Selenoprotein P	BPI fold-containing family B member 1	Vitronectin
101	Vimentin	Actin-related protein 2	Serine/cysteine peptidase inhibitor, clade C member 1	Placenta-specific 8
102	T-kininogen 1	40S ribosomal protein S3a	Uncharacterized protein	Complement C1q subcomponent subunit B

103	Elongation factor 1-delta	Serine/cysteine peptidase inhibitor, clade C member 1	Eukaryotic peptide chain release factor GTP-binding subunit ERF3B-like	BPI fold-containing family B member 1
104	Septin-9	Retinal dehydrogenase 1	Ig lambda-2 chain C region	Uncharacterized protein
105	T-complex protein 1 subunit beta	Myosin, heavy polypeptide 9	Activator of Hsp90 ATPase activity 1	Serine/cysteine peptidase inhibitor, clade C (Antithrombin), member 1
106	Complement C3	Inter-alpha trypsin inhibitor, heavy chain 1	40S ribosomal protein S25	Heat shock protein HSP 90-alpha
107	RCG31562, isoform CRA_c	Ube2l3 protein	40S ribosomal protein S5	Poly(rC)-binding protein 2
108	Uncharacterized protein	Actin-related protein 2/3 complex subunit 4	Serotransferrin	40S ribosomal protein S25
109	Myristoylated alanine-rich C-kinase substrate	C4b-binding protein alpha chain	Calcium-binding protein 4	**Pulmonary surfactant-associated protein A**
110	Similar to 60S acidic ribosomal protein P2	Adiponectin c	Complement C3	RAB5C
111	Rps16 protein	Actin-related protein 3	Vitronectin	Immunoglobulin joining chain
112	**Pulmonary surfactant-associated protein B**	**Pulmonary surfactant-associated protein D**	Uncharacterized protein	Heat shock cognate 71 kDa protein
113	Vitronectin	Plasminogen	60S ribosomal protein L22	Complement C3
114	Adenine phosphoribosyltransferase	40S ribosomal protein S25	Protein disulfide-isomerase	Uncharacterized protein
115	Uncharacterized protein	Small ubiquitin-related modifier 2	RAB1A	40S ribosomal protein S7
116	Fibronectin	Coronin-1A	Rps16 protein	Complement component C9
117	Dynein light chain 1	Prothrombin	40S ribosomal protein S6	Microsomal glutathione S-transferase
118	Calpain small subunit 1	Uncharacterized protein	40S ribosomal protein S14	Histone H4
119	Murinoglobulin-1	Immunoglobulin joining chain	Histone H2B	Rps16 protein
120	Barrier-to-autointegration factor	60S ribosomal protein L5	Plasminogen activator inhibitor 1 RNA-binding protein	Protein S100-A4

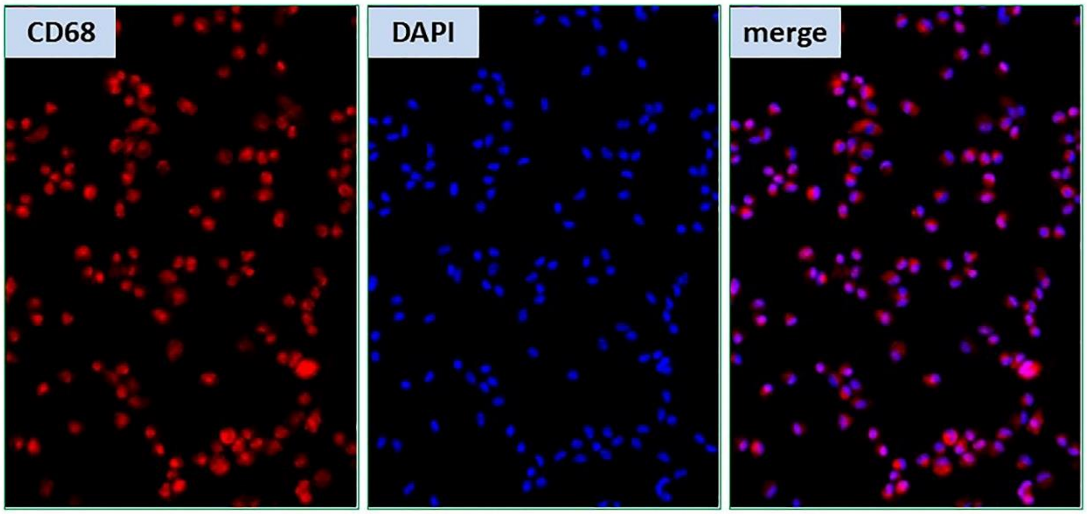

Figure S5 Immunostaining of primary alveolar macrophages

Isolated primary alveolar macrophages were immunostained with CD68, a macrophage marker, to confirm their identity. The nucleus was stained with DAPI. The imagines were observed and captured using a florescence microscope (ZEISS LSM880+Airyscan, Germany)

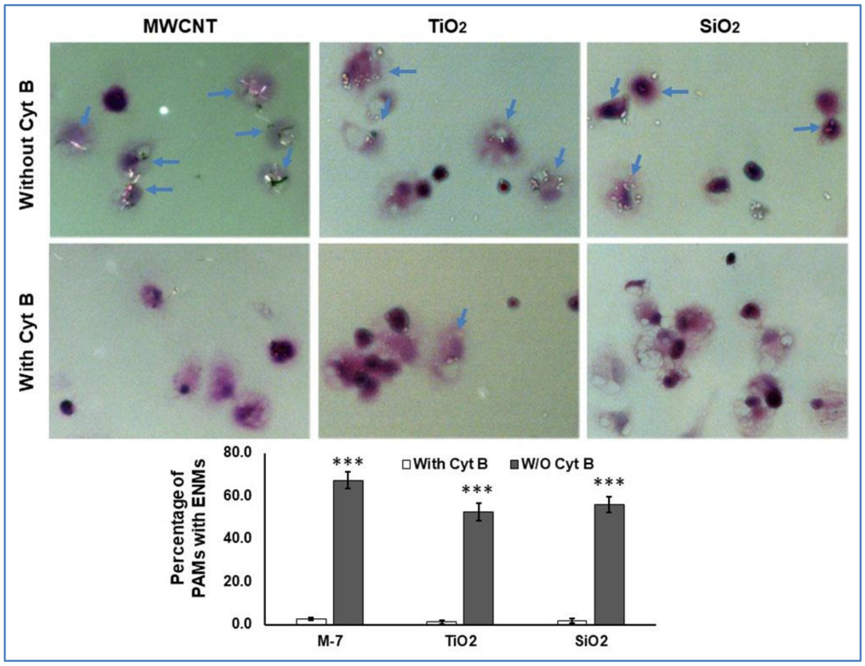

Figure S6 Uptake of ENMs by PAMs via phagocytosis

5×10^5 primary alveolar macrophages (PAMs) in a 6-well plate were treated with or without 5μg/ml cytochalasin B (Cyt B, an inhibitor of actin polymerization, Solarbio, Beijing, China) for 1h and then exposed with MWCNT-7, TiO_2, or SiO_2 pre-incubated with BALF for another 12h. The cells were fixed, eosin-stained and observed under an ECLIPSE polarizing microscope (LV100NPOL, Nikon, Japan). Percentages of the PAMs with burden particles (arrows) were calculated.

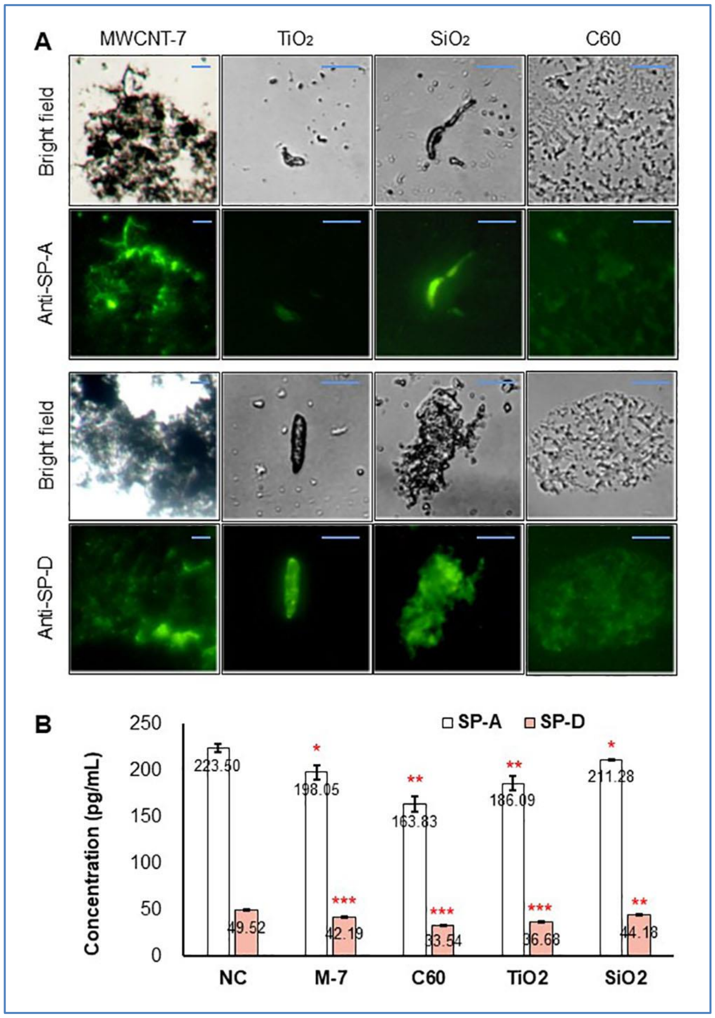

Figure S7 Binding of SP-A and SP-D to the ENMs

400 μl of each of 4 ENM suspensions (equal to 200 μg of each ENM) was incubated with 250 μl of the 10-fold concentrated BALF at 37℃ in a shaker at 200 rpm for 6 hours, centrifuged at 4℃, 20000 × g, for 10 min. A. the precipitated ENMs were used for immunofluorescence staining and the supernatants were used for ELISA detection of SP-A and SP-D. Briefly, the precipitates were washed 3 times and incubated with rabbit anti-rat SP-A or SP-D antibodies (1:100 dilution, Affinity, Wuhan, China) at 4℃ overnight. After washing, the ENMs were visualized with Cy3 labelled goat anti-rabbit IgG (1:100 dilution, Proteintech, Wuhan, China). Images were captured with a florescence microscope (ZEISS LSM880+Airyscan, Germany). B. SP-A and SP-D in the supernatants were detected using rat ELISA kits (MLBio, Shanghai, China) according to the manufacturer's instructions. Two-tailed students' t-test was used for statistical comparison, and *, ** and *** represent p values less than 0.05, 0.01 and 0.001, respectively.

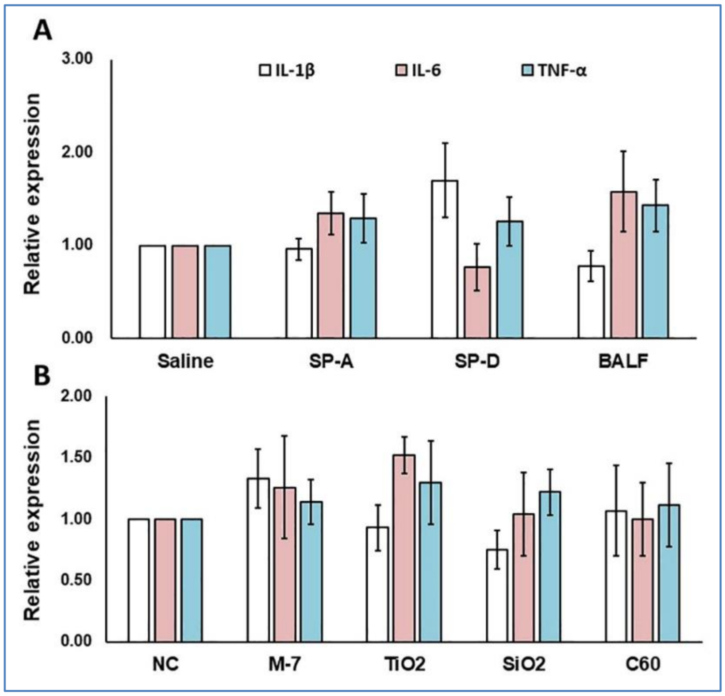

Figure S8 Effects of SP-A, SD-D, BALF and ENMs on expression of cytokines

1×10^6 primary alveolar macrophages (PAMs) in a 6-well plate were treated with saline, 1μg/ml SP-A/SP-D, or 20μl of 10-fold concentrated BALF for 12h (A); 1×10^6 PAMs were treated with saline containing 0.5% PF-68 (NC), 1μg/ml of MWCNT-7, TiO_2, SiO_2, or C60 suspensions for 12h (B). Total RNAs were isolated for qRT-PCR analysis of IL-1β, TNF-α and IL-6, using GAPDH as an internal reference. Two-tailed students' t-test was used for statistical comparison.

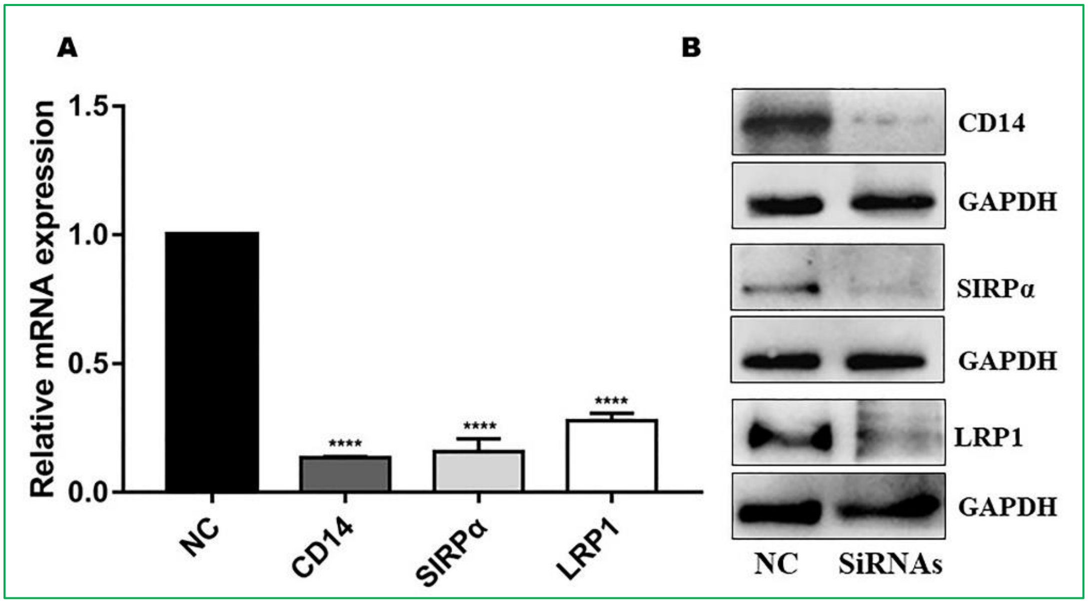

Figure S9 Knockdown of CD14, LRP1, and SIRPα expression in PAMs

CD14, LRP1, and SIRPα expression in primary alveolar macrophages was knocked down by gene-specific siRNAs. The silencing efficacy for LRP1, CD14, and SIRPα was analyzed by qPCR (A) and confirmed by western blotting (B).

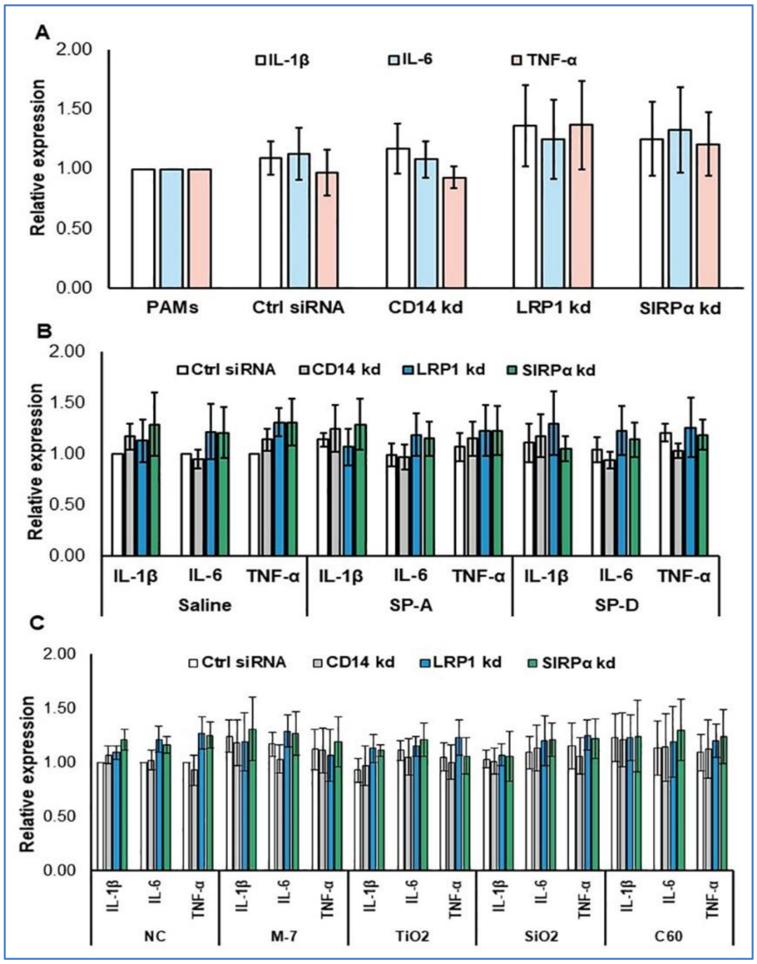

Figure S10 Effects of SP-A, SD-D, BALF and ENMs on expression of cytokines in the receptors-knocked down PAMs

1×10^6 rat PAMs in a 6-well plate was transfected with negative control RNA, or gene-specific siRNAs for LRP1, CD14, and SIRPα and cultured at 37℃ for 6h (A); and after the culture media was changed to X-VIVO^{TM} serum-free medium (Lonza), the cells were treated with saline, 1μg/ml of SP-A or SP-D and cultured for 12h (B), or treated with saline containing 0.5% (NC), 1μg/ml of MWCNT-7, TiO_2, SiO2, or C60 suspended in saline containing 0.5% for 12h. Total RNAs were isolated for qRT-PCR analysis of IL-1β, TNF-α and IL-6, using GAPDH as an internal reference. Two-tailed students' t-test was used for statistical comparison.

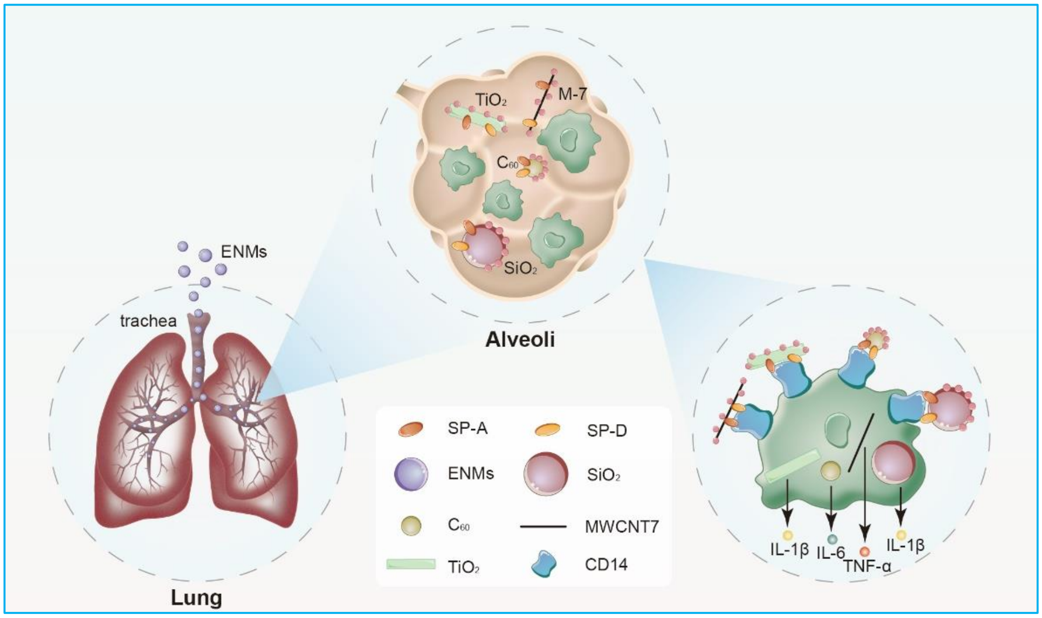

Figure 11 Summarized results
